# Inflammatory monocytes promote progression of Duchenne muscular dystrophy and can be therapeutically targeted via CCR2

**DOI:** 10.15252/emmm.201403967

**Published:** 2014-10-13

**Authors:** Kamalika Mojumdar, Feng Liang, Christian Giordano, Christian Lemaire, Gawiyou Danialou, Tatsuma Okazaki, Johanne Bourdon, Moutih Rafei, Jacques Galipeau, Maziar Divangahi, Basil J Petrof

**Affiliations:** 1 Meakins-Christie Laboratories and Respiratory Division, McGill University Health Centre and Research InstituteMontreal, QC, Canada; 2 Department of Pharmacology, Faculty of Medicine, University of MontrealMontreal, QC, Canada; 3 Department of Hematology and Oncology, Winship Cancer Institute, Emory UniversityAtlanta, GA, USA; 4 Department of Pediatrics, Emory UniversityAtlanta, GA, USA

**Keywords:** CCR2, chemokines, inflammatory monocytes, macrophage polarization, muscular dystrophy

## Abstract

Myofiber necrosis and fibrosis are hallmarks of Duchenne muscular dystrophy (DMD), leading to lethal weakness of the diaphragm. Macrophages (MPs) are required for successful muscle regeneration, but the role of inflammatory monocyte (MO)-derived MPs in either promoting or mitigating DMD is unclear. We show that DMD (mdx) mouse diaphragms exhibit greatly increased expression of CCR2 and its chemokine ligands, along with inflammatory (Ly6C^high^) MO recruitment and accumulation of CD11b^high^ MO-derived MPs. Loss-of-function of CCR2 preferentially reduced this CD11b^high^ MP population by impeding the release of Ly6C^high^ MOs from the bone marrow but not the splenic reservoir. CCR2 deficiency also helped restore the MP polarization balance by preventing excessive skewing of MPs toward a proinflammatory phenotype. These effects were linked to amelioration of histopathological features and increased muscle strength in the diaphragm. Chronic inhibition of CCR2 signaling by mutated CCL2 secreted from implanted mesenchymal stem cells resulted in similar improvements. These data uncover a previously unrecognized role of inflammatory MOs in DMD pathogenesis and indicate that CCR2 inhibition could offer a novel strategy for DMD management.

## Introduction

Duchenne muscular dystrophy (DMD), the most common X-linked lethal disorder in humans, is caused by defects of the gene encoding dystrophin, a 427 kDa cytoskeletal protein found at the inner surface of the skeletal muscle surface membrane. The binding between dystrophin and its associated glycoprotein complex confers important structural and signal transduction properties to the muscle fiber (Petrof, 2002), the absence of which leads to muscle fiber necrosis that is followed by progressive fibrosis. The diaphragm and other respiratory muscles are also subjected to this process, and most patients with DMD die of respiratory muscle failure in early adulthood unless artificially supported by mechanical ventilation. Furthermore, in the naturally occurring murine model of DMD (the mdx mouse), which also lacks dystrophin, the diaphragm is severely affected from an early age and closely resembles the human phenotype with respect to relentless fiber loss, fibrosis, and reduced muscle strength (Stedman *et al*, 1991).

The lack of dystrophin in skeletal muscles leads to multiple cellular defects, such as increased mechanical fragility of the muscle fiber membrane, increased calcium entry into the cell, and dysregulated nitric oxide signaling (Petrof, 2002). In addition, activated NF-κB and proinflammatory genes are upregulated within DMD muscles from shortly after birth (Chen *et al*, 2005). This early inflammatory response is followed in later stages by a failure of muscle regeneration with associated muscle fibrosis. In mdx mice, blockade of the NF-κB pathway (Messina *et al*, 2006; Acharyya *et al*, 2007) or inflammatory mediators such as TNF-α and inducible nitric oxide synthase (iNOS) (Radley *et al*, 2008; Villalta *et al*, 2009) reduces muscle necrosis, suggesting that the host inflammatory response plays an important role in promoting muscle injury and subsequent fibrosis.

Myeloid cells of the monocyte/macrophage (MO/MP) lineage constitute by far the predominant inflammatory cell type within DMD and mdx muscles (Spencer *et al*, 2001; Wehling *et al*, 2001). MOs originate from the bone marrow and traffic to peripheral tissues, where they differentiate into MPs with different functional characteristics (Ingersoll *et al*, 2011; Shi & Pamer, 2011). In mice, the cell surface marker Ly6C identifies a MO subset expressing high levels of CC chemokine receptor 2 (CCR2), which binds members of the monocyte chemoattractant protein (MCP) family of CC chemokines that are critically involved in MO/MP recruitment (Geissmann *et al*, 2003). In the setting of acute infectious or sterile tissue injury, Ly6C^high^ (often referred to as ‘inflammatory’) MOs exit the bone marrow under the regulation of CCR2 (Ingersoll *et al*, 2011). Upon entry into tissues, these cells differentiate into MPs which may then become polarized toward a ‘classical inflammatory’ or alternatively activated ‘anti-inflammatory/wound-healing’ phenotype, depending upon complex environmental and other influences that are as yet poorly understood (Mosser & Edwards, 2008; Martinez *et al*, 2009; Sica & Mantovani, 2012). Excessive skewing toward inflammatory MPs in dystrophic muscles would be expected to increase their cytolytic effects upon muscle fibers and thus promote necrosis (Villalta *et al*, 2009). On the other hand, an overabundance of anti-inflammatory/wound-healing MPs can favor the development of muscle fibrosis (Vidal *et al*, 2008).

It is well established that inhibition of CCR2 signaling reduces MP accumulation and impedes successful regeneration in normal muscles that are acutely injured (Warren *et al*, 2005; Contreras-Shannon *et al*, 2007; Sun *et al*, 2009). This is consistent with data indicating that MPs produce trophic factors which play an important supportive role in the repair and functional recovery of normal skeletal muscle (Saclier *et al*, 2013). However, the latter situation differs greatly from dystrophic muscles, which are characterized by a state of smoldering inflammation with chronically increased MP accumulation (Porter *et al*, 2002). It is unknown whether these abnormally abundant MPs are derived from CCR2-dependent blood-borne inflammatory Ly6C^high^ MOs or a non-hematopoietic lineage of MPs that normally gives rise to the resident MP population (Schulz *et al*, 2012). In either case, the sustained MP infiltration of dystrophic muscles could be maladaptive and thus contribute to disease progression (Vidal *et al*, 2008; Villalta *et al*, 2009).

In the present study, we sought to determine whether and how CCR2 inhibition might influence the recruitment pattern of inflammatory MOs and polarization features of MPs in dystrophic muscles, as well as the impact upon overall disease severity. Here, we demonstrate for the first time that in diametric opposition to the situation observed in previously healthy muscles subjected to injury, loss of CCR2 function in dystrophic mice (either genetically or pharmacologically induced) improves characteristic histopathological features of the disease and more importantly substantially increases the force-generating capacity of the diaphragm. CCR2 deficiency in mdx mice preferentially reduced the CD11b^high^ population of MPs derived from recently recruited Ly6C^high^ inflammatory MOs and promoted recovery of normal MP polarization characteristics. These findings represent the first direct evidence of a critical role for CCR2-driven inflammatory MO recruitment in DMD pathogenesis and additionally identify CCR2 as an important modulator of disease severity, thus indicating its potential to serve as a new therapeutic target in DMD.

## Results

### Upregulation of CCR2 and its ligands in dystrophic muscles

**Figure 1:**
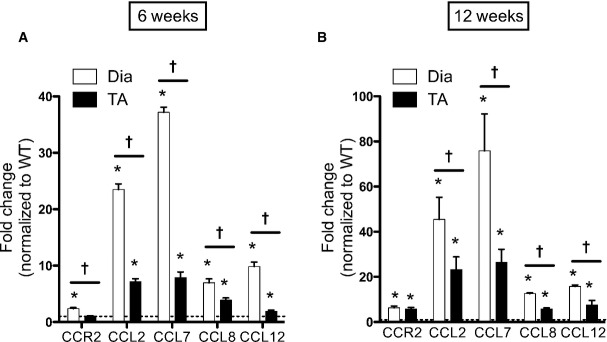
Upregulation of CCR2 and its chemokine ligands in dystrophic muscles Transcript levels of CCR2 and its ligands in diaphragm (DIA) and tibialis anterior (TA) muscles of mdx mice. Data are expressed as fold change compared to age-matched WT controls; the dashed line represents WT expression levels for each gene (*n* = 9 mice per group, three independent experiments).At 6 weeks of age, asterisk (*) indicates significant difference between WT and mdx as follows: *P* = 8.2e-6 (for DIA CCR2), *P* = 1.2e-13 (for DIA CCL2), *P* = 5.8e-9 (for TA CCL2), *P* = 2.8e-17 (for DIA CCL7), *P* = 5.3e-6 (for TA CCL7), *P* = 3.6e-7 (for DIA CCL8), *P* = 2.9e-5 (for TA CCL8), *P* = 7.7e-9 (for DIA CCL12), *P* = 0.001 (for TA CCL12); cross (^†^) indicates significant difference between DIA and TA as follows: *P* = 0.0003 (for CCR2), *P* = 8.1e-7 (for CCL2), *P* = 2.2e-7 (for CCL7), *P* = 0.008 (for CCL8), *P* = 2.2e-6 (for CCL12).At 12 weeks of age, * comparison between WT and mdx as follows: *P* = 2.5e-6 (for DIA CCR2), *P* = 7.5e-6 (for TA CCR2), *P* = 0.0004 (for DIA CCL2), *P* = 0.001 (for TA CCL2), *P* = 0.0003 (for DIA CCL7), *P* = 0.0005 (for TA CCL7), *P* = 8e-14 (for DIA CCL8), *P* = 2.3e-7 (for TA CCL8), *P* = 3.9e-14 (for DIA CCL12), *P* = 0.004 (for TA CCL12); ^†^ comparison between DIA and TA as follows: *P* = 0.049 (for CCL2), *P* = 0.008 (for CCL7), *P* = 5.3e-9 (for CCL8), *P* = 0.004 (for CCL12).

Because the diaphragm exhibits the most severe dystrophic features in mdx mice, we compared the mRNA expression levels of CCR2 and its ligands (CCL2, CCL7, CCL8, CCL12) in mdx diaphragm and limb muscle (tibialis anterior, TA). This was done at 6 weeks (Fig 1A) and 12 weeks (Fig 1B); the younger age represents the acute inflammatory stage of the disease, whereas older age group muscles have entered a less intense chronic inflammatory phase. In both age groups, mdx diaphragms demonstrated CCR2 expression levels which were significantly increased above WT values (elevated 2.4-fold at 6 weeks and 6.3-fold at 12 weeks). In the TA limb muscle, CCR2 expression was similarly upregulated at 12 weeks (5.8-fold increase over WT).

The upregulation of CCR2 ligand expression in mdx muscles was even more prominent than of the CCR2 receptor. This was particularly striking for CCL2 and CCL7, which were greatly increased above WT at 6 weeks (23.5-fold and 37.2-fold, respectively), and even more so at 12 weeks (45.5-fold and 75.9-fold, respectively) in the mdx diaphragm. Importantly, both CCL2 and CCL7 have been shown to play critical roles in the mobilization of MOs from bone marrow to blood (Tsou *et al*, 2007). A similar pattern of CCR2 ligand upregulation was also observed in the mdx TA muscle, albeit at a lower level than in the diaphragm. Taken together, these data show that CCR2 and its associated chemokine ligands are greatly elevated in dystrophic mdx muscles during both the acute and the chronic inflammatory stages of the disease.

### CCR2 deficiency reduces macrophage accumulation in dystrophic muscle

**Figure 2:**
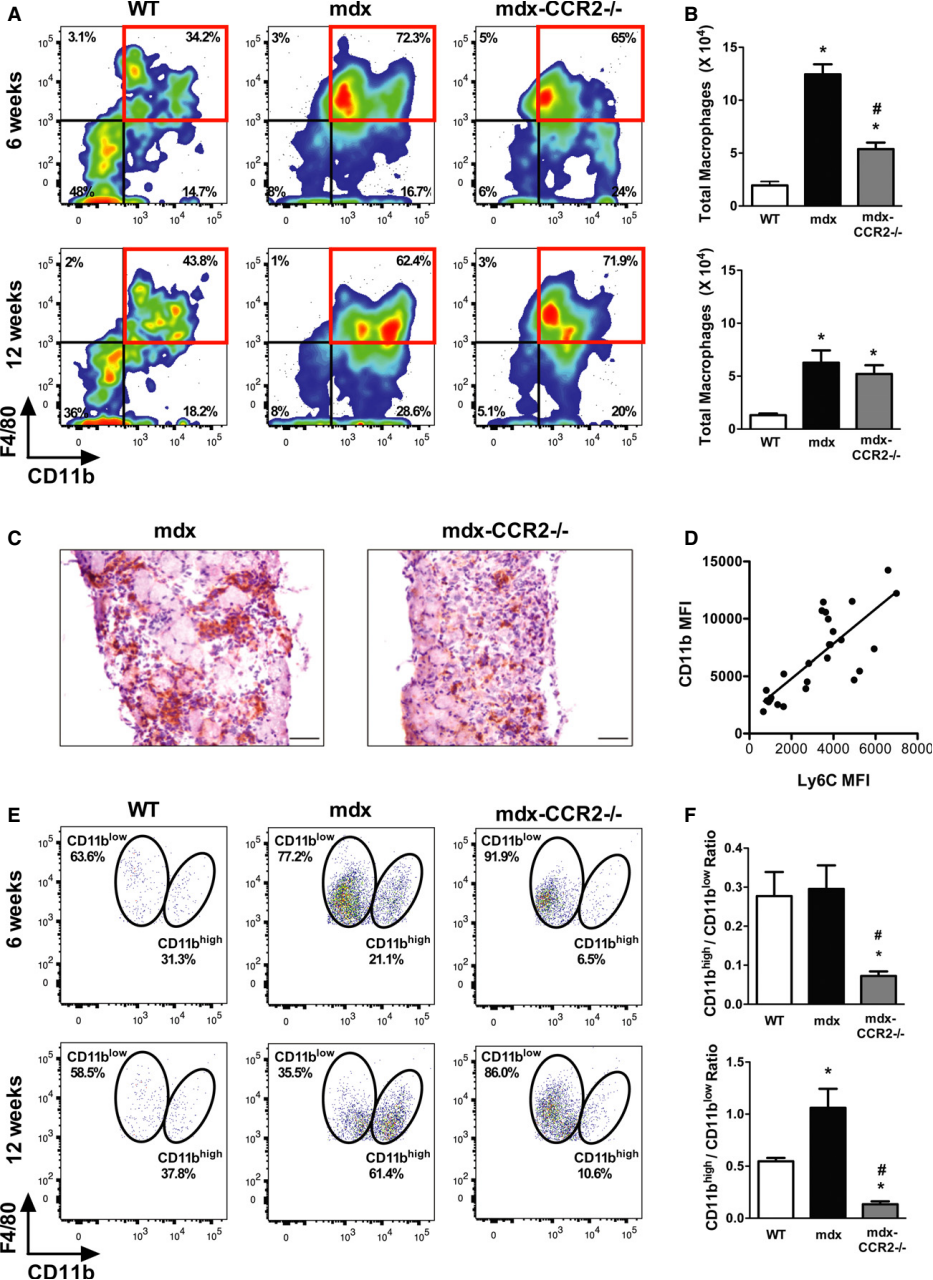
CCR2 deficiency reduces macrophage accumulation in dystrophic diaphragms Representative flow cytometry plots of CD45^+^ cells from WT, mdx, and mdx-CCR2^−/−^ groups at 6 weeks (left panel) and 12 weeks (right panel), showing percentages of infiltrating MPs in the diaphragm.Total numbers of diaphragm MPs (CD45^+^CD11b^+^F4/80^+^) in the three mouse strains. For 6 weeks of age (upper panel), * comparison with WT: *P* = 1.3e-8 (for mdx) and *P* = 0.006 (for mdx-CCR2^−/−^); ^#^ comparison between mdx and mdx-CCR2^−/−^: *P* = 2.7e-6. For 12 weeks of age (lower panel), * comparison with WT: *P* = 0.001 (for mdx) and *P* = 0.01 (for mdx-CCR2^−/−^).Infiltrating MPs detected by F4/80 immunostaining (brown staining) in mdx (left panel) and mdx-CCR2^−/−^ (right panel) mice at 6 weeks of age. Magnification 20×, scale bars represent 50 μm.Correlation (*r* = 0.79, *P* = 5.2e-7) between mean fluorescent intensities (MFI) of Ly6C and CD11b on diaphragm MPs from combined WT, mdx, and mdx-CCR2^−/−^ at 6 weeks and 12 weeks.Representative flow cytometry dot plots of MPs classified as CD11b^high^ and CD11b^low^ based on CD11b expression level.Ratio of CD11b^high^ to CD11b^low^ MPs in the three mouse strains; data are group means ± SE (*n* = 7 mice per group, three independent experiments). At 6 weeks (upper panel), * comparison to WT: *P* = 0.034; ^#^ comparison between mdx and mdx-CCR2^−/−^: *P* = 0.022. For 12 weeks of age (lower panel), * comparison to WT: *P* = 0.01 (for mdx) and *P* = 0.046 (for mdx-CCR2^−/−^); ^#^ comparison between mdx and mdx-CCR2^−/−^: *P* = 0.0001.

To evaluate the role of CCR2 and its ligands in muscular dystrophy pathogenesis, we generated CCR2-deficient mdx mice (mdx-CCR2^−/−^). Flow cytometry was performed on cell suspensions from the entire diaphragm muscle, and intramuscular MPs were identified on the basis of their prototypical markers CD11b and F4/80 (Fig 2A and Supplementary Fig S1). Mdx mice contained higher numbers of intramuscular MPs than WT mice, and this was particularly evident at 6 weeks of age (Fig 2B and C). The mdx-CCR2^−/−^ mice demonstrated a group mean of 57% reduction in overall MP numbers (as well as a 40% reduction of CD45^+^ cells) relative to the mdx group at 6 weeks, whereas no significant difference in total MP numbers or CD45^+^ cells was found between these two groups at 12 weeks.

Recent work has provided evidence for the existence of two major MP lineages in mice: a hematopoietic MO-derived lineage which is the major contributor to CD11b^high^ tissue MPs and a non-hematopoietic embryonic yolk sac-derived lineage which gives rise to CD11b^low^ resident tissue MPs (Schulz *et al*, 2012). We found a strong correlation between the mean fluorescent intensities of Ly6C and CD11b expression in MPs extracted from muscle (Fig 2D), consistent with the CD11b^high^ MPs being derived from recently recruited Ly6C^high^ inflammatory MOs. Furthermore, the great majority (over 90%) of Ly6C^high^ MOs in the blood expressed CCR2 (Supplementary Fig S2). A closer analysis of intramuscular MPs within the diaphragm revealed discernible CD11b^high^ and CD11b^low^ subpopulations (Fig 2E). In mdx mice, the numbers of both CD11b^high^ and CD11b^low^ MPs were increased compared to WT muscles. Most strikingly, in mdx-CCR2^−/−^ mice, the ratio of CD11b^high^ to CD11b^low^ MPs was greatly reduced not only relative to the mdx group, but also in comparison with WT mice (Fig 2F). This was found to be true during both the acute (6 weeks) and the chronic (12 weeks) inflammatory stages of the disease. The findings are thus consistent with a scenario in which CCR2 deficiency preferentially inhibits intramuscular accumulation of the recruited, blood-derived MO/MP population.

**Figure 3:**
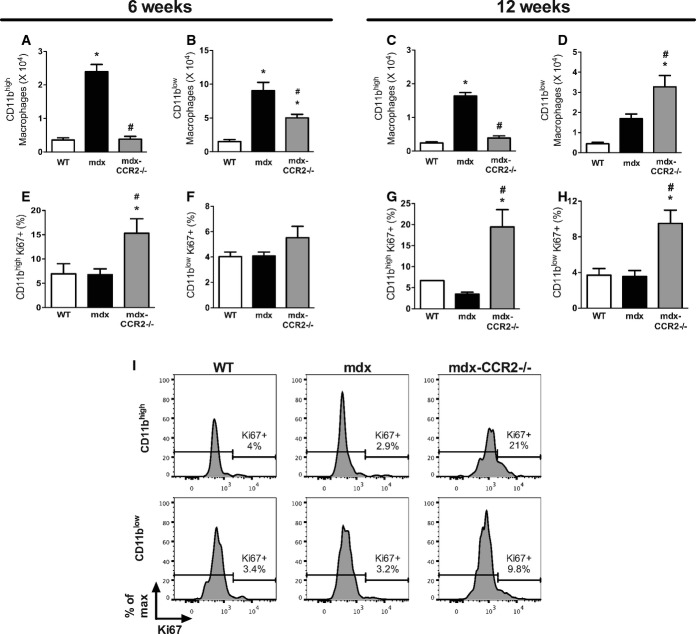
Effects of CCR2 deficiency on macrophage proliferation in dystrophic muscles Absolute number of CD11b^high^ MPs in the diaphragm at 6 weeks, * comparison to WT: *P* = 6.1e-7, ^#^ comparison between mdx and mdx-CCR2^−/−^: *P* = 7.1e-7.Absolute number of CD11b^low^ MPs at 6 weeks, * comparison to WT: *P* = 6.6e-5 (for mdx) and *P* = 0.02 (for mdx-CCR2^−/−^), ^#^ comparison between mdx and mdx-CCR2^−/−^: *P* = 0.01.Absolute counts of CD11b^high^ MPs in the diaphragm at 12 weeks, * comparison to WT: *P* = 9.1e-8, ^#^ comparison between mdx and mdx-CCR2^−/−^: *P* = 6.1e-7.Absolute number of CD11b^low^ MPs at 12 weeks, * comparison to WT: *P* = 0.0003, ^#^ comparison between mdx and mdx-CCR2^−/−^: *P* = 0.02.Percentage of proliferating (Ki67^+^) cells among CD11b^high^ MPs in the three mouse strains at 6 weeks, * comparison to WT: *P* = 0.03, ^#^ comparison between mdx and mdx-CCR2^−/−^: *P* = 0.02.Percentage of Ki67^+^ proliferating cells in CD11b^low^ MPs at 6 weeks.Percentage of Ki67^+^ cells in CD11b^high^ MPs at 12 weeks, * comparison to WT: *P* = 0.002, ^#^ comparison between mdx and mdx-CCR2^−/−^: *P* = 0.0002.Percentage of proliferating cells in CD11b^low^ MPs at 12 weeks, * comparison to WT: *P* = 0.02, ^#^ comparison between mdx and mdx-CCR2^−/−^: *P* = 0.02.Representative histograms demonstrating Ki67^+^ proliferating CD11b^high^ and CD11b^low^ MPs at 12 weeks. Data information: Data are group means ± SE (*n* = 5 (A–D), 6 (E, F) and 7 (G, H) mice per group; three independent experiments).

Figure3A–D show the absolute numbers of CD11b^high^ and CD11b^low^ MPs present in diaphragms of the three mouse strains. In comparison with mdx, absolute numbers of CD11b^high^ MPs in the mdx-CCR2^−/−^ group were significantly reduced at both 6 weeks (6.2-fold) and 12 weeks (4.2-fold) of age. However, while CD11b^low^ MPs were decreased at 6 weeks in the mdx-CCR2^−/−^ group (1.8-fold), there was actually a twofold increase of CD11b^low^ MPs in these mice at 12 weeks. Differences in MP numbers within the muscle could potentially be related to altered levels of MP recruitment, survival, or proliferation. To determine whether lack of CCR2-dependent MO/MP recruitment affects the local proliferation rate of CD11b^high^ and CD11b^low^ MPs in dystrophic muscle, Ki67 staining was evaluated on these MP subpopulations (gating shown in Supplementary Fig S3). As demonstrated in Fig 3E–I, mdx-CCR2^−/−^ mice muscles contained a significantly higher percentage of Ki67^+^ cells for both CD11b^high^ (at 6 and 12 weeks) and CD11b^low^ (at 12 weeks) MPs, relative to the WT and mdx groups. Therefore, despite a major reduction in recruited MPs in mdx-CCR2^−/−^ compared to mdx mice, there is a higher level of local intramuscular MP proliferation in the former group.

### CCR2 deficiency alters inflammatory monocyte compartmental distribution in dystrophic mice

**Figure 4:**
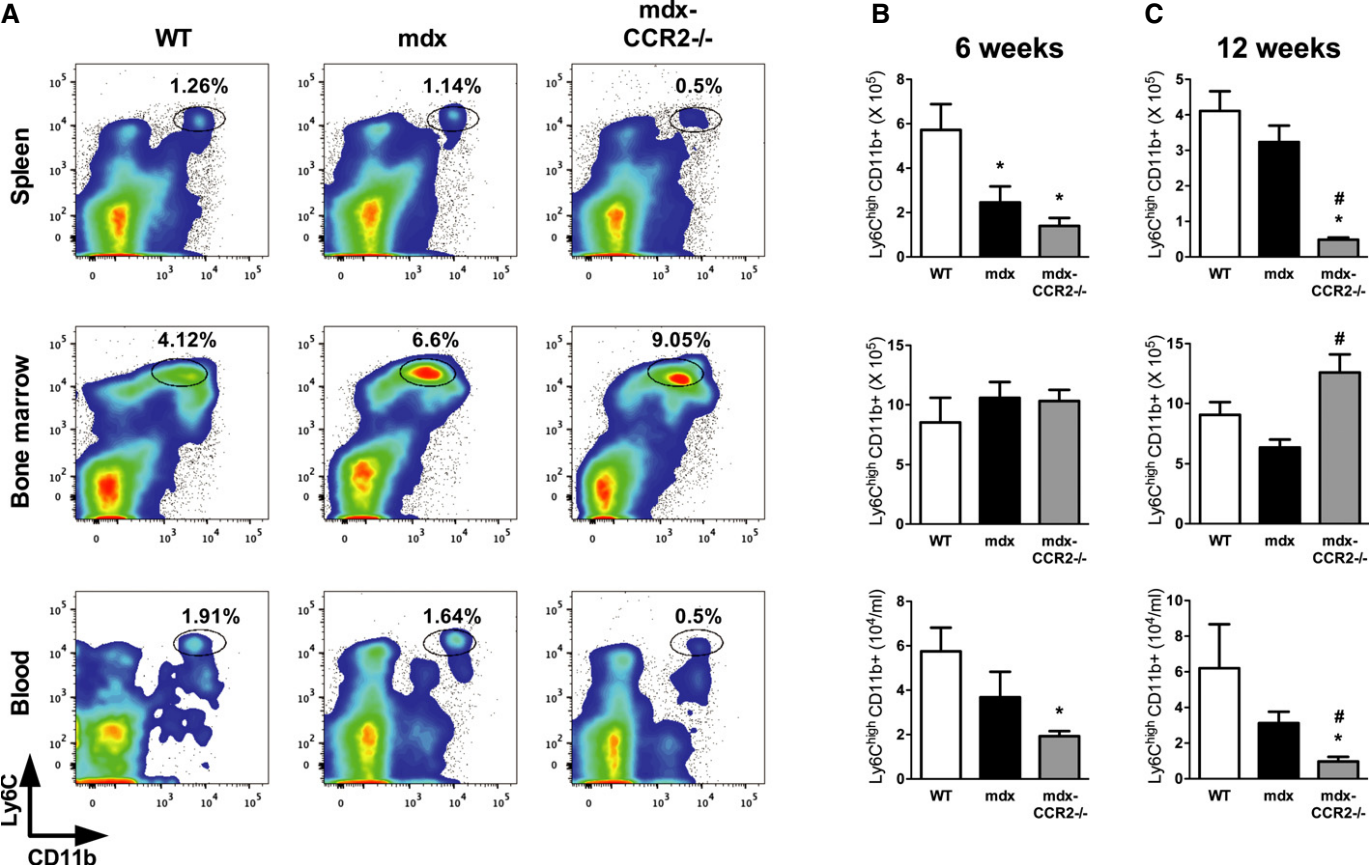
Effects of CCR2 deficiency on inflammatory monocyte dynamics in non-muscle tissues of dystrophic mice Representative flow cytometry plots of CD45^+^ cells from WT, mdx, and mdx-CCR2^−/−^ groups at 6 weeks, showing percentages of Ly6C^high^ CD11b^+^ MOs within spleen, bone marrow (expressed per femur), and blood.Total number of MOs at 6 weeks in spleen (upper panel), * comparison to WT: *P* = 0.03 (for mdx) and *P* = 0.004 (for mdx-CCR2^−/−^); bone marrow (middle panel); and blood (lower panel), * comparison to WT: *P* = 0.02.Absolute MO counts in the three mouse strains at 12 weeks in spleen (upper panel), * comparison to WT: *P* = 0.0001, ^#^ comparison between mdx and mdx-CCR2^−/−^: *P* = 0.002; in bone marrow (middle panel), ^#^ comparison between mdx and mdx-CCR2^−/−^: *P* = 0.006; and in blood (lower panel), * comparison to WT: *P* = 0.002, ^#^ comparison between mdx and mdx-CCR2^−/−^: *P* = 0.008. Data information: Data are group means ± SE (*n* = 5 mice per group, three independent experiments).

**Figure 5:**
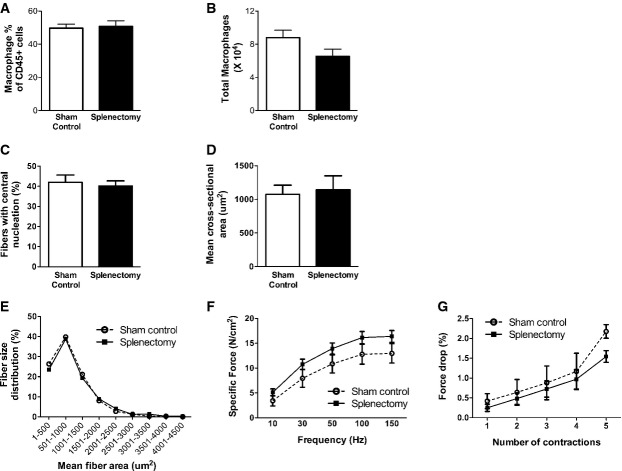
Splenic monocytes do not contribute to early dystrophic pathology Quantification of MPs as a percentage of CD45^+^ leukocytes in control and splenectomized groups.Total diaphragm MP number quantification.Percentage of centrally nucleated fibers in diaphragms of control and splenectomized animals.Mean cross-sectional area of centrally nucleated diaphragm fibers.Diaphragm fiber size distribution.Force–frequency curves during electrical stimulation of diaphragm muscle bundles.Eccentric contraction-induced force drop of diaphragm muscle from the two groups. There were no significant differences between control and splenectomized groups for any of the above parameters. Data information: Data are group means ± SE (control *n *=* *6, splenectomy *n *=* *7; three independent experiments).

Previous work has shown that inflammatory Ly6C^high^ MOs which are able to infiltrate into injured tissues and convert to MPs can be mobilized into the bloodstream from both bone marrow and splenic reservoirs (Geissmann *et al*, 2003; Arnold *et al*, 2007; Nahrendorf *et al*, 2007; Swirski *et al*, 2009). To examine this question in more detail, we determined the frequency of inflammatory Ly6C^high^ MO/MPs in the splenic, bone marrow, and blood compartments by flow cytometry (Fig 4 and Supplementary Fig S4). At 6 weeks of age in both mdx and mdx-CCR2^−/−^ mice, the absolute numbers of Ly6C^high^ MO/MPs within the spleen were significantly reduced compared to WT mice, suggesting that the splenic reservoir might have been called upon to supply these cells to injured dystrophic muscles. This persisted at 12 weeks in mdx mice lacking CCR2, and increased numbers of Ly6C^high^ MOs in the bone marrow at this time as well as reduced MOs in the blood also indicated the possibility of impaired bone marrow exit as previously described in CCR2-deficient mice (Serbina & Pamer, 2006). Because spleen-derived MO/MPs can play a role in tissue injury or healing (Swirski *et al*, 2009), we next performed surgical removal of the spleen at 3 weeks of age, immediately prior to onset of the acute inflammatory phase in mdx mice. This did not significantly impact upon the level of MPs in the muscle (Fig 5A and B) or the development of dystrophic histopathology and impaired diaphragmatic strength 6 weeks later (Fig 5C–G). Accordingly, these data suggest that spleen-derived MO/MPs do not play a major role in modulating early disease evolution in this model.

### CCR2-deficient mdx mice are shifted away from exaggerated inflammatory macrophage polarization

**Figure 6:**
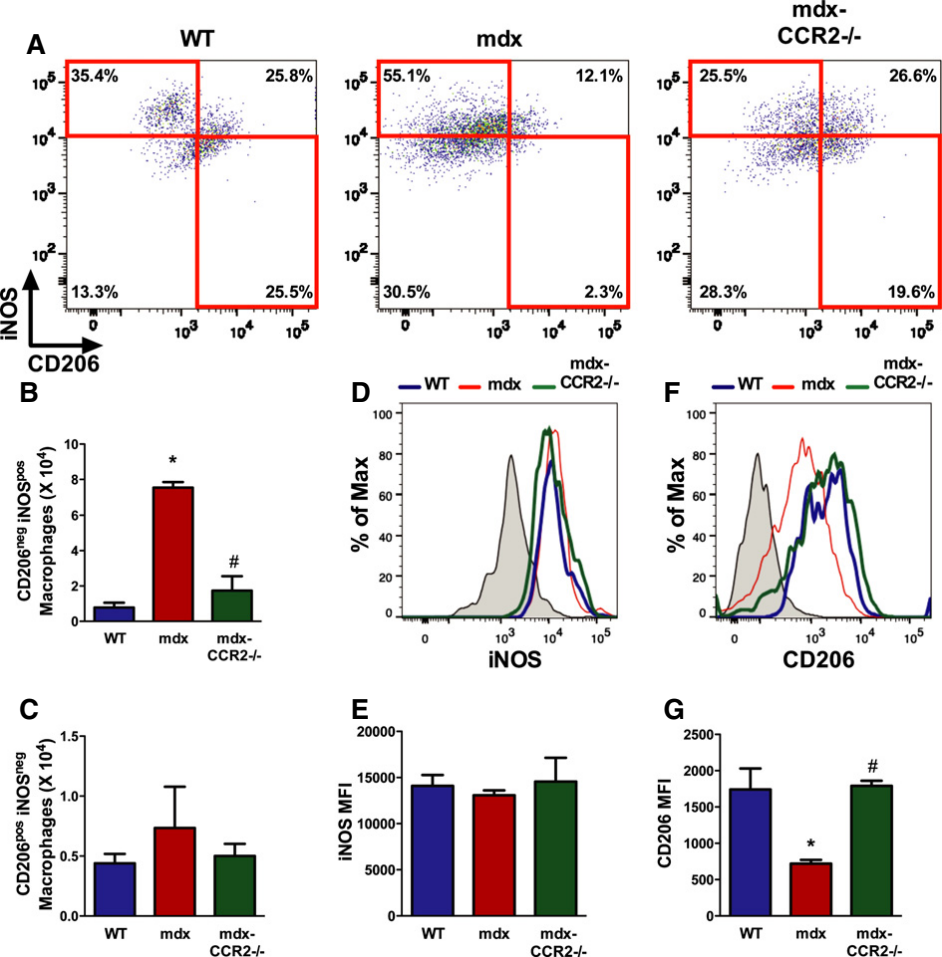
CCR2-deficient dystrophic diaphragms exhibit reduced inflammatory macrophage polarization Flow cytometry was employed to assess iNOS and CD206 expression in CD11b^+^F4/80^+^ macrophages (MPs) at 6 weeks.Representative dot plots showing diaphragm MPs with CD206^neg^ iNOS^pos^ and CD206^pos^ iNOS^neg^ profiles in WT, mdx, and mdx-CCR2^−/−^ groups.Quantification of total numbers of CD206^neg^ iNOS^pos^ MPs in the diaphragm, * comparison to WT: *P *=* *6.5e–5, ^#^ comparison between mdx and mdx-CCR2^−/−^: *P *=* *0.0002.Quantification of total numbers of CD206^pos^ iNOS^neg^ MPs in the diaphragm.Overlay histograms of iNOS expression in the three groups. Shaded curve represents isotype control.iNOS median fluorescence intensity (MFI) quantification on diaphragm MPs.Overlay histograms of CD206 expression in the three groups.CD206 MFI quantification on diaphragm MPs, * comparison to WT: *P* = 0.005, ^#^ comparison between mdx and mdx-CCR2^−/−^: *P* = 0.004. Data information: All data are group means ± SE (WT and mdx: *n* = 4; mdx-CCR2^−/−^: *n* = 3; two independent experiments).

It has been reported that Ly6C^high^ inflammatory MOs released into the blood enter into injured muscles and initially undergo differentiation into phagocytic inflammatory MPs, which later switch their phenotype to an anti-inflammatory (alternative) profile that favors muscle regeneration (Arnold *et al*, 2007). In addition to blunting the overall accumulation of MPs within dystrophic diaphragms during the acute inflammatory phase of the disease, we posited that the balance between inflammatory and anti-inflammatory MPs could also be altered by CCR2 deficiency. We assessed this aspect of MP phenotype by flow cytometry (Fig 6A and Supplementary Fig S5), using the classical MP polarization markers iNOS and CD206 (Mantovani *et al*, 2004).

In mdx mice, CD206^neg^ iNOS^pos^ MPs were significantly increased in number compared to WT mice (Fig 6A and B). Conversely, the percentage of CD206^pos^ iNOS^neg^ was decreased in mdx mice, but their absolute number (Fig 6C) remained similar to the WT group by virtue of the fact that mdx diaphragms contained a much higher total number of MPs. In the mdx-CCR2^−/−^ group, there was a 4.3-fold reduction in the number of CD206^neg^ iNOS^pos^ MPs relative to the mdx group (Fig 6B), whereas the total number of CD206^pos^ iNOS^neg^ did not differ (Fig 6C). Because many of the cells did not clearly segregate into these two discrete populations (particularly in mdx and mdx-CCR2^−/−^ mice), we also evaluated the mean fluorescent intensities of iNOS and CD206 expression (Fig 6D–G) in all MPs. This analysis revealed that CD206 expression in MPs was significantly reduced in mdx compared to WT, but recovered to normal values in the mdx-CCR2^−/−^ group (Fig 6F and G). Taken together, the above findings suggest that CCR2 ablation in mdx mice significantly restores the normal intramuscular MP polarization balance.

### CCR2 deficiency reduces dystrophic histopathology

**Figure 7:**
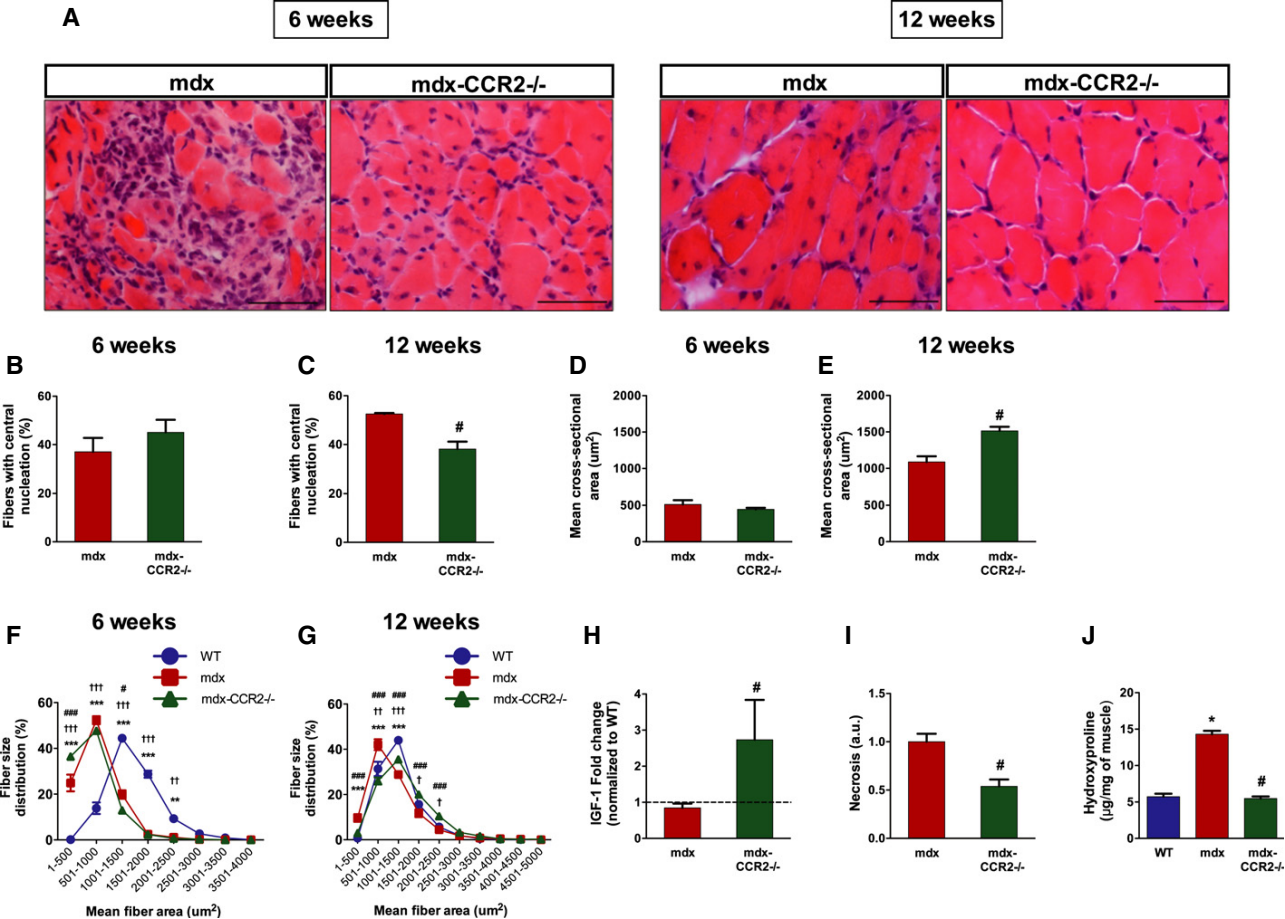
Genetic ablation of CCR2 decreases dystrophic diaphragm histopathology A Representative images of transverse diaphragm sections stained with hematoxylin and eosin at 6 weeks (left panel) and 12 weeks (right panel) in mdx and mdx-CCR2^−/−^ mice (scale bars represent 50 μm). B, C Percentage of fibers with central nucleation at (B) 6 weeks and (C) 12 weeks, ^#^*P* = 8.7e-6. D, E Mean cross-sectional area of centrally nucleated fibers at (D) 6 weeks and (E) 12 weeks, ^#^*P* = 0.002. F, G Fiber size distribution in WT, mdx, and mdx-CCR2^−/−^ diaphragms at (F) 6 weeks and (G) 12 weeks, **P < 0.01, ***P < 0.001 for WT compared to mdx; ^†^*P* < 0.05, ^††^*P* < 0.01, ^†††^*P* < 0.001 for WT compared to mdx-CCR2^−/−^; ^#^*P* < 0.05, ^##^*P* < 0.01, ^###^*P* < 0.001 for mdx compared to mdx-CCR2^−/−^. H–J In diaphragms at 12 weeks: (H) insulin-like growth factor (IGF)-1 mRNA levels (dashed line represents WT level), ^#^*P* = 0.004; (I) muscle necrosis quantification expressed relative to mdx, ^#^*P* = 0.001; and (J) hydroxyproline content, * comparison to WT: *P* = 1.7e-8, ^#^ comparison between mdx and mdx-CCR2^−/−^: *P* = 1e-8. Data information: Data are group means ± SE (6 weeks: WT *n* = 5, mdx *n* = 5, mdx-CCR2^−/−^ *n* = 6; 12 weeks: WT *n* = 5, mdx *n* = 5, mdx-CCR2^−/−^ *n* = 7; three independent experiments).

Since intramuscular MP accumulation and excessive skewing (in either direction) of the MP polarization balance have been implicated in muscular dystrophy pathogenesis, we next determined whether CCR2 deficiency affected the severity of muscle disease. A hallmark of DMD is the presence of regenerated muscle fibers containing centrally located nuclei (see Fig 7A), which are almost entirely absent in WT mice and can thus be used as a semi-quantitative marker for prior necrosis-regeneration cycles in mdx muscles (Karpati *et al*, 1988). At 6 weeks of age, there was no difference between mdx and mdx-CCR2^−/−^ groups in the percentage of centrally nucleated regenerated fibers (Fig 7B). However, by 12 weeks of age, centrally nucleated fibers were less prevalent in mdx-CCR2^−/−^ diaphragms (Fig 7C). Furthermore, the average (mean) cross-sectional area of centrally nucleated fibers (Fig 7D and E) was larger in the mdx-CCR2^−/−^ mice at 12 weeks, suggesting more effective reconstitution of regenerated fiber size in this group. A size distribution analysis for all fibers (Fig 7F and G) confirmed an overall shift toward larger fiber sizes in mdx-CCR2^−/−^ diaphragms as well as increased expression of the regeneration-promoting growth factor IGF-1 (Fig 7H). In addition, IgG staining and hydroxyproline assays indicated reduced necrosis (Fig 7I) and decreased collagen content (Fig 7J), respectively, in the mdx-CCR2^−/−^ group in comparison with mdx mice at 12 weeks of age. Interestingly, bone marrow-derived MPs from mdx-CCR2^−/−^ mice also expressed lower osteopontin and higher MMP2/9 mRNA levels (Supplementary Fig S6), which is consistent with a less fibrogenic phenotype (Vetrone *et al*, 2009; Onozuka *et al*, 2011). Similar improvements in dystrophic pathology were also found in the TA muscles of mdx-CCR2^−/−^ mice (Supplementary Fig S7). The above findings collectively suggest that CCR2 ablation reduced cycles of necrosis while at the same time promoting more effective muscle repair with less associated fibrosis.

### Force-generating capacity is increased in CCR2-deficient mdx mice

**Figure 8:**
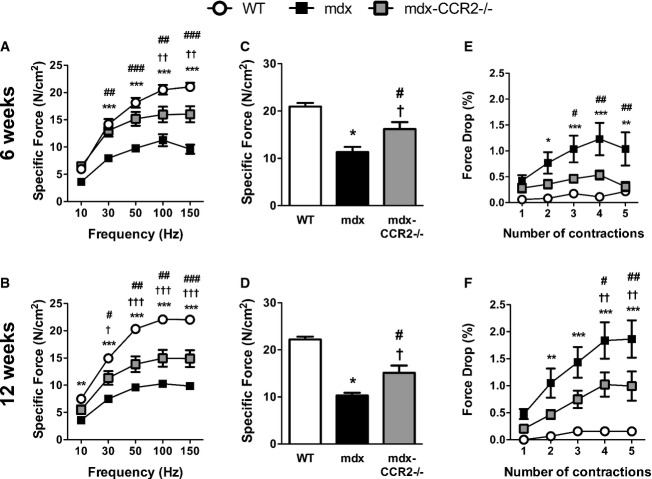
CCR2 ablation improves force-generating capacity of the dystrophic diaphragm A, B Force–frequency curves during electrical stimulation of diaphragms from WT, mdx, and mdx-CCR2^−/−^ mice at (A) 6 weeks and (B) 12 weeks. C, D Quantification of maximum force-generating capacity of diaphragms at (C) 6 weeks and (D) 12 weeks. E, F Eccentric contraction-induced force drop quantification for the diaphragm at (E) 6 weeks and (F) 12 weeks. Data information: In panels (A–B) and (E–F): *P < 0.05, **P < 0.01, ***P < 0.001 for WT compared to mdx; ^†^*P* < 0.05, ^††^*P* < 0.01, ^†††^*P* < 0.001 for WT compared to mdx-CCR2^−/−^; ^#^*P* < 0.05, ^##^*P* < 0.01, ^###^*P* < 0.001 for mdx compared to mdx-CCR2^−/−^. For panel (C): * comparison between WT and mdx: *P* = 1.8e-5; ^†^ comparison between WT and mdx-CCR2^−/−^: *P* = 0.02; ^#^ comparison between mdx and mdx-CCR2^−/−^: *P* = 0.02. For panel (D): * comparison between WT and mdx: *P* = 2.1e-8; ^†^ comparison between WT and mdx-CCR2^−/−^: *P* = 1.2e-5, ^#^ comparison between mdx and mdx-CCR2^−/−^: *P* = 0.01. Data are expressed as mean ± SE (6 weeks: *n* = 8 per group; 12 weeks: WT *n* = 8, mdx *n* = 7, mdx-CCR2^−/−^ *n* = 6; three independent experiments).

To determine whether the above histopathological amelioration translated into functional improvements, we evaluated the force-generating capacity of the diaphragm by electrical stimulation *ex vivo*. As anticipated, mdx diaphragms generated less force than WT at both 6 weeks (Fig 8A) and 12 weeks (Fig 8B) of age. Importantly, the diaphragms of mdx-CCR2^−/−^ mice showed significant improvements in maximal force-generating capacity at both ages, amounting to average increases of 43 and 47% over mdx values at 6 weeks (Fig 8C) and 12 weeks (Fig 8D), respectively. In addition, we determined the extent of the force drop resulting from the repeated imposition of eccentric contractions, a method used for evaluating the ability of dystrophic muscles to resist contraction-induced mechanical damage (Petrof *et al*, 1993; Dudley *et al*, 2006). CCR2 deficiency significantly reduced the magnitude of the contraction-induced force drop in mdx diaphragms at both 6 weeks (Fig 8E) and 12 weeks (Fig 8F). Therefore, in addition to improved muscle histology, genetic ablation of CCR2 conferred significant functional benefits upon the muscles of mdx mice.

### Pharmacological blockade of CCR2 is beneficial in mdx mice

**Figure 9:**
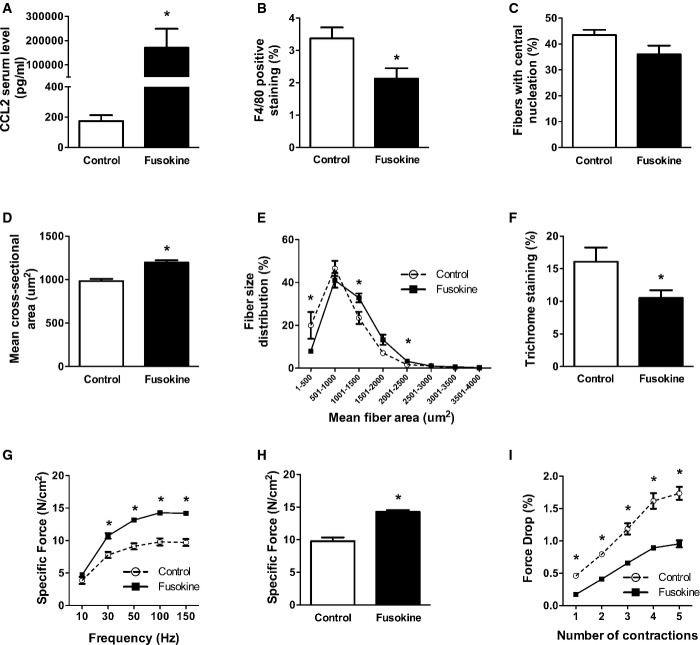
Pharmacological blockade with an anti-CCR2 fusokine ameliorates the dystrophic diaphragm phenotype At 6 weeks after initiation of treatment, comparisons between fusokine (*n* = 7) and sham (*n* = 5) treated mice are shown for:Fusokine protein (truncated CCL2) levels in serum, *P = 3.8e-9.Diaphragm MP infiltration, *P = 0.03.Percentage of centrally nucleated diaphragm fibers.Mean cross-sectional area of centrally nucleated fibers, *P = 0.0002.Fiber size distribution, *P = 0.03 (for 1–500 μm^2^), *P* = 0.02 (for 1,001–1,500 μm^2^), and *P* = 0.02 (for 2,001–2,500 μm^2^).Fibrosis quantification, *P = 0.049.Force–frequency curves during electrical stimulation of the diaphragm, *P = 0.0009 (for 30 Hz), *P* = 2.7e-5 (for 50 Hz), *P* = 1.2e-5 (for 100 Hz), and *P* = 8.8e-6 (for 150 Hz).Maximal force-generating capacity of the diaphragm, *P = 1.1e-5.Force drop in the diaphragm following eccentric contractions, *P = 3.1e-5 (for contraction 1), *P* = 4.7e-6 (for contraction 2), *P* = 9.2e-5 (for contraction 3), *P* = 6.3e-5 (for contraction 4), and *P* = 2.5e-5 (for contraction 5). Data information: Data are group means ± SE from three independent experiments.

Finally, to assess the potential for pharmacologic intervention, we treated mdx mice with a CCR2-inhibiting ‘fusokine’ molecule (truncated CCL2/GM-CSF fusion protein) which was secreted systemically by retrovirally engineered mesenchymal stromal cells (Rafei *et al*, 2009, 2011) implanted subcutaneously into mdx mice at 3 weeks of age. High levels of the fusokine molecule were measured in the serum at 6 weeks after initiation of the treatment (Fig 9A), and there was a decrease in MP infiltration of the diaphragm (Fig 9B). At this time in the diaphragms of fusokine- versus sham-treated mdx mice, there was a trend (*P* = 0.098) toward reduced percentage of centrally nucleated fibers (Fig 9C), a significant increase in the size of centrally nucleated fibers (Fig 9D), a shift in overall fiber size distribution toward larger fibers (Fig 9E), and reduced fibrosis (Fig 9F). In addition, the diaphragms of fusokine-treated mdx mice exhibited improvements in maximal force-generating capacity (Fig 9G and H) and greater resistance to contraction-induced mechanical damage (Fig 9I). Therefore, pharmacological blockade of CCR2 at an early stage of the disease resulted in a significant amelioration of the dystrophic phenotype in a manner which was similar to that observed in the genetic CCR2 ablation model.

## Discussion

DMD is the most common X-linked lethal disorder in humans, with an incidence of approximately 1 in 3,500 male births. Absence of the dystrophin protein leads to muscle fiber death, principally through necrosis. Because the disease involves the diaphragm and other respiratory muscles, most patients die of respiratory failure by their early 20s unless supported by mechanical ventilation. At the present time, there is very little in the way of effective therapy. Although the ideal treatment would be gene therapy to restore dystrophin expression to all muscles of the body, major biological and technical hurdles will first need to be overcome. In the meanwhile, there is an urgent need for new therapies. Corticosteroids are the only agents known to slow disease progression, but with very limited success and considerable side effects (Manzur *et al*, 2008).

The results of this study reveal a key role for CCR2-dependent inflammatory MO/MP recruitment in promoting disease progression in dystrophin-deficient skeletal muscle. We thus show for the first time that interference with CCR2 function, through either genetic or pharmacologic means, confers substantial therapeutic benefits to mdx mice. At the tissue level, mdx-CCR2^−/−^ diaphragms exhibited improved force generation, increased fiber cross-sectional area, and reduced fibrosis. These beneficial effects upon the disease were associated with two principal effects upon MO/MP behavior. Firstly, CCR2 deficiency resulted in a major attenuation of inflammatory MO recruitment to dystrophic muscles. Secondly, MPs within the muscles of CCR2-deficient dystrophic mice exhibited a less proinflammatory phenotype. Taken together, these results suggest that CCR2-dependent chemokine signaling to MO/MPs is critically involved in driving pathological inflammation and tissue injury in DMD.

In DMD muscles, the composition of the inflammatory infiltrate, that is, a predominance of MPs, is consistent with the observed upregulation of CCR2 and its ligands. CCR2 binds to several chemokine ligands including CCL2 (MCP-1), CCL8 (MCP-2), CCL7 (MCP-3), CCL13 (MCP-4), and CCL12 (MCP-5). Interestingly, the benefits of blocking CCR2 which we observed in mdx mice are in marked contrast to the situation in acutely injured healthy muscles, where intact CCR2 signaling is essential for effective muscle repair. The latter is highlighted by studies showing that ablation of either CCR2 or CCL2 results in delayed muscle regeneration and impaired muscle strength recovery (Martinez *et al*, 2010; Contreras-Shannon *et al*, 2007; Shireman *et al*, 2007). Although we and others have shown that myoblasts and their precursors (satellite cells) can express CCR2 and respond to CCL2 stimulation *in vitro* (Warren *et al*, 2005; Yahiaoui *et al*, 2008), bone marrow chimera models have indicated that CCR2 signaling in the myeloid lineage rather than myogenic cells is the main prerequisite for successful regeneration of normal muscles following acute injury (Sun *et al*, 2009).

It is important to recognize that dystrophic muscles present an entirely different scenario, which is one of the chronic repetitive injuries with smoldering inflammation. The lack of a concerted switch toward a more anti-inflammatory MP phenotype in mdx mice is in marked contrast to what occurs in acute muscle injury models (Arnold *et al*, 2007; Lu *et al*, 2011), thus reiterating the differences between self-limited inflammation following acute injury of normal muscle and the incomplete resolution of inflammation which is characteristic of muscular dystrophy (Porter *et al*, 2002). In this setting, much of the tissue damage and subsequent fibrotic response of DMD skeletal muscle appears to be driven by the host inflammatory response (Messina *et al*, 2006; Acharyya *et al*, 2007; Villalta *et al*, 2009).

In our study, the decreased prevalence of centrally nucleated (regenerated) fibers observed in CCR2-deficient mdx mice is consistent with a reduction in necrosis-regeneration cycles (Karpati *et al*, 1988). Furthermore, the fact that regenerated fibers were larger in mdx-CCR2^−/−^ diaphragms and the reduced level of fibrosis both suggest that mdx muscle fiber repair processes were more effective in the absence of CCR2. Anti-inflammatory MPs in skeletal muscle are known to produce muscle growth-promoting mediators such as IGF-1 (Arnold *et al*, 2007; Lu *et al*, 2011). In addition, although these ‘wound-healing’ type MPs can either favor (Vidal *et al*, 2008) or inhibit (Pesce *et al*, 2009) tissue fibrosis depending upon the specific context, our data suggest that the tissue injury induced by proinflammatory mediators released from inflammatory MPs (Villalta *et al*, 2009) is likely to be an important early initiator of muscle fibrosis in mdx mice. Inflammatory cytokines and reactive oxygen species can also directly suppress force production in the absence of detectable histological injury (Whitehead *et al*, 2008; Bellinger *et al*, 2009; Piers *et al*, 2011), and reduced exposure to such proinflammatory mediators is another probable mechanism for improved force generation in mdx-CCR2^−/−^ diaphragms.

The cross talk between injured skeletal muscles and recruited inflammatory cells is mediated to a large extent by chemokines such as CCL2 and CCL7, which have important effects in mobilizing MOs from the bone marrow (Tsou *et al*, 2007). These chemokine ligands of CCR2 are substantially elevated not only within dystrophic muscles but also in the serum of mdx mice (Nelson *et al*, 2011). In mice, at least two distinct subsets of blood MOs are known to exist. The Ly6C^low^ subset exhibits low CCR2 expression and appears to mostly play a homeostatic patrolling function within the circulation (Auffray *et al*, 2007). On the other hand, Ly6C^high^ MOs express high levels of CCR2 and are rapidly recruited to sites of acute infection or inflammation, where they give rise to inflammatory MPs (Arnold *et al*, 2007). Accordingly, the decrease of inflammatory profile MPs within the muscles of mdx-CCR2^−/−^ mice can most readily be explained by two factors. First, the release of Ly6C^high^ MOs from the bone marrow is reported to be significantly impeded in mice lacking CCR2 (Serbina & Pamer, 2006; Lu *et al*, 2011), thus accounting for reduced transit of these cells into the bloodstream. Second, for Ly6C^high^ MOs that do achieve exit from the bone marrow, their ability to leave the circulation and gain entrance to injured muscles is additionally impaired by the absence of CCR2 (Serbina & Pamer, 2006; Lu *et al*, 2011).

The mechanisms governing MP fate and polarization in skeletal muscle are only beginning to be understood (Saclier *et al*, 2013). In several non-muscle tissues, it was recently reported that murine MPs are derived from two major sources: a CD11b^high^ hematopoietic lineage derived from blood-borne cells and a CD11b^low^ non-hematopoietic lineage originating from the embryonic yolk sac that gives rise to the resident MP population (Schulz *et al*, 2012). Although this issue has not to our knowledge been specifically examined in skeletal muscle, we found a strong correlation between expression levels of Ly6C and CD11b in intramuscular MPs. This is consistent with the concept that CD11b^high^ MPs are primarily derived from recently recruited Ly6C^high^ inflammatory MOs. Importantly, CCR2 deficiency preferentially reduced the accumulation of these CD11b^high^ MPs during both the acute and the chronic stages of inflammation in dystrophic muscles.

In addition to inflammatory MO recruitment as a source of intramuscular MPs, the MP population within tissues is able to proliferate and expand itself locally (Jenkins *et al*, 2013; Hashimoto *et al*, 2013; Robbins *et al*, 2013; Yona *et al*, 2013). In the present study, we found that CCR2 deficiency increased the local proliferation of MPs in dystrophic muscles, initially in the recruited CD11b^high^ population (at 6 weeks) and later in both CD11b^high^ and CD11b^low^ MPs (at 12 weeks). This suggests that a significant proportion of the accumulated MPs within mdx-CCR2^−/−^ muscles may have originated from local proliferation and helps to explain the fact that total MP numbers did not differ between mdx and mdx-CCR2^−/−^ mice at 12 weeks. Our observations are also consistent with a recent report that clodronate-treated rats (depleted of monocytes) and then subjected to experimental injury, also showed increased proliferation of both inflammatory and anti-inflammatory profile MPs within the affected muscles (Côté *et al*, 2013). Taken together, the above findings collectively suggest significant adaptive compensation via local MP proliferation for the reduced MO/MP recruitment in dystrophic mice lacking CCR2.

Our findings in the dystrophic mouse model are reminiscent of murine experimental autoimmune encephalitis (EAE), in which blockade or genetic deletion of CCR2 also reduces inflammatory MO/MP accumulation and disease severity (Izikson *et al*, 2000; Mildner *et al*, 2009). In other experimental models characterized by abnormal inflammatory skewing of MPs such as diabetes (Nio *et al*, 2012) and obesity (Lumeng *et al*, 2007), CCL2 or CCR2 deficiency has also been linked to a switch away from excessive proinflammatory MP polarization. In the EAE model, the initial activation of resident tissue MPs (microglia) is believed to be an important early trigger for the accumulation of MPs derived from inflammatory MOs (Ajami *et al*, 2011). Resident MPs within injured skeletal muscle have also been reported to be a major source of CCL2 (Brigitte *et al*, 2010) and could thus serve as a trigger for CCR2-driven MO recruitment to dystrophic muscles. In addition, although CCR2 deficiency does not affect the numbers of B or T cells within acutely injured muscles (Lu *et al*, 2011), we do not exclude a possible role for altered regulatory T-cell (Burzyn *et al*, 2013) or Th2-type T-lymphocyte responses (Traynor *et al*, 2000; Kim *et al*, 2001), which could in turn lead to the production of cytokines such as IL-4 and IL-13 that promote anti-inflammatory MP polarization (Sica & Mantovani, 2012). The recruitment of blood-borne fibrocytes implicated in tissue fibrosis is also CCR2 responsive (Moore *et al*, 2005) and could theoretically play a role in the beneficial effects of CCR2 inhibition observed in our study.

In summary, the results of this study reveal that CCR2 is an important driver of persistent inflammation and progressive muscle degeneration in dystrophin-deficient muscles. Interference with CCR2 function in this context not only reduces the absolute numbers of recruited MO/MPs within dystrophic muscles, but also mitigates the exaggerated inflammatory skewing of MPs that can provoke further tissue injury and impede muscle regeneration (Saclier *et al*, 2013). Pharmacological agents that inhibit CCR2 signaling are already showing promise for other diseases in both humans (Gilbert *et al*, 2011) and preclinical animal models (Qian *et al*, 2011). Although corticosteroids have potent anti-inflammatory properties and are currently the only approved pharmacological therapy for DMD, these drugs have substantial side effects and low specificity, which likely explains their poor efficacy (Manzur *et al*, 2008). The results of this study offer the prospect of a novel therapy for DMD with more specific and targeted anti-inflammatory actions.

## Materials and Methods

### Animals

All animal procedures were approved by the institutional animal care and ethics committee, in accordance with the guidelines issued by the Canadian Council on Animal Care. Wild-type (WT; C57BL/10ScSn/J), mdx (C57BL10ScSn-*mdx*/J), and CCR2^−/−^ (B6.129S4-Ccr2^tm1Ifc^/J, backcrossed for nine generations to C57BL/6) mice were originally purchased from The Jackson Laboratories (Bar Harbor, ME). Male CCR2^−/−^ mice were crossed with homozygous mdx females to produce male mdx mice that were heterozygous for the CCR2 mutation; these animals were then bred with homozygous mdx females to create both male and female mdx mice heterozygous for the CCR2 mutation. The latter were used to generate mdx mice homozygous for the CCR2 mutation (mdx-CCR2^−/−^) on a mixed BL10 (75%) and BL6 (25%) background, which were used for subsequent experiments. It should be noted that previous work indicates no differences in inflammation, fibrosis or myofiber damage in mdx mice on the BL10 and BL6 backgrounds (Beastrom *et al*, 2011), and we also found no differences in macrophage infiltration or force generation between BL10 and BL6 mice (Supplementary Fig S8). Mice were screened for the dystrophin mutation by PCR as previously described (Amalfitano & Chamberlain, 1996). To identify CCR2-deficient mice, the PCR reaction (94°C 1 min; 65°C 1.5 min; 72°C 1.5 min; for 35 cycles) used two primer sets amplifying a portion of the wild-type CCR2 allele and the neomycin cassette (see Supplementary Table S1 for primer sequences). The animals were euthanized at either 6 or 12 weeks of age with removal of both the diaphragm and tibialis anterior (TA), the latter being a frequently studied but less severely impaired limb muscle in mdx mice.

### Flow cytometry

Diaphragm infiltrating MPs were characterized and quantified in WT, mdx, and mdx-CCR2^−/−^ animals at 6 weeks and 12 weeks of age. Diaphragm, blood, bone marrow, and spleen were harvested. Single cell suspensions were obtained from entire diaphragms (excluding the central tendon) by mincing the muscle into small pieces in ice-cold PBS. The muscle pieces were incubated in buffered 0.2% collagenase B (Roche) solution for 1 h at 37°C followed by filtering of the cell suspension through a 70-μm cell strainer. Bone marrow cells were harvested by flushing both femurs and tibiae with ice-cold PBS. Spleens were ground and passed through 70-μm cell strainers to obtain single cell suspensions. Total viable cell numbers harvested from each organ were determined by Trypan blue stain exclusion and were used to calculate the absolute counts of various cell populations. Following red cell lysis, the cells were resuspended in FACS buffer (PBS with 0.5% BSA) and pre-incubated in blocking solution (anti-CD16/CD32, BD Biosciences). The cells were then surface stained using the following fluorescently labeled antibodies for 20 min at 4°C: VF00-labeled anti-mouse CD45 (clone 30-F11, BD Biosciences), Alexa Fluor 488-labeled anti-mouse CD11b (clone M1/70), PE-Cy7-labeled anti-mouse F4/80 (clone BM8), and Alexa Fluor 647-labeled anti-mouse Ly6C or with PE-labeled anti-mouse Ly6C (clone HK1.4; all from BioLegend) and APC-labeled anti-mouse CCR2 (clone # 475301, R&D Systems).

To assess MP proliferation, diaphragm cells were surface stained for VF00-labeled anti-mouse CD45 (BD Biosciences), APC-Cy7-labeled anti-mouse CD11b, and PE-Cy7-labeled anti-mouse F4/80 (all from BioLegend). Cells were washed, fixed, and permeabilized using Fox-P3 buffer set (BD Pharmingen), followed by staining for intra-nuclear Ki67 (clone SolA 15, FITC-labeled, eBioscience). To characterize MP polarization phenotypes, diaphragm cells were similarly surface stained for CD45, CD11b, F4/80, Ly6C, and CD206 (clone C068C2) (all from BioLegend). Stained cells were washed, fixed in 4% PFA, permeabilized using PBS/0.3% Triton, and then stained intracellularly with FITC-labeled anti-mouse iNOS (clone 6/iNOS/NOS Type II, BD Biosciences). Cells were washed to remove excess antibodies, resuspended in FACS buffer and acquired on a BD LSR-II. For each sample, 100,000–500,000 events were recorded. Data analysis was done using FlowJo software (Treestar Inc., Ashland, OR, USA).

Cells were gated based on their forward and side scatter properties, followed by gating on CD45^+^ leukocytes. MPs in diaphragm were characterized as CD11b^+^F4/80^+^ cells and were further classified as CD11b^high^ or CD11b^low^ MPs based on the level of CD11b expression on non-permeabilized cells. To identify proliferating MPs, intra-nuclear Ki67 expression was assessed in CD11b^high^ and CD11b^low^ permeabilized MPs. For MP polarization analysis, CD11b and F4/80 were used to identify intramuscular MPs, followed by gating CD206^neg^ iNOS^pos^ and CD206^pos^ iNOS^neg^ MPs. Inflammatory MOs in bone marrow, blood, and spleen were defined as CD11b^+^ cells with high expression of Ly6C. Appropriate isotype controls were used to set negative population gates.

### Splenectomy

Animals were assigned to the sham and splenectomized groups randomly. At 3 weeks of age, mdx mice were anesthetized by isoflurane gas anesthesia, and a laparotomy was performed under sterile conditions. The spleen was gently removed after surgical ligature of its vascular supply. For the sham-operated control group, laparotomy was performed under identical conditions but without removal of the spleen. Animals were allowed to recover from surgery and then euthanized 6 weeks later for analysis.

### Real-time PCR quantification of gene expression

Total RNA was extracted from tissues or cells using TRIzol reagent (Invitrogen, USA) according to the manufacturer's protocol. RNA was treated with DNase I (Gibco, USA), purified using the RNeasy minikit (Qiagen, Germany), and then quantified by spectrophotometric optical density measurement. The purified RNA was reverse-transcribed to cDNA with random primers and SuperScript II (Invitrogen, USA) reverse transcriptase. Quantitative RT–PCR was performed using 5 ng of cDNA mixed with 10 μl SYBR® Green Master Mixes (SABiosciences, USA) and 1 μl of 10 μM primer mixes. RT–PCR was carried out for 40 cycles at a melting temperature of 95°C for 15 s and an annealing temperature of 60°C for 1 min using a StepOne Plus Thermocycler (Applied Biosystems, USA). Mouse HPRT1 was used as an internal control. The relative quantification of gene expression was analyzed by the 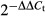 method, and the results are expressed as n-fold difference relative to control. Primer sequences are shown in Supplementary Table S1.

### Histological analysis

Cryostat tissue sections obtained from the mid-portion of each muscle were stained with hematoxylin and eosin. Randomly selected microscopic fields from each tissue section were then photographed using a QImaging Retiga-2000R digital camera mounted on an Olympus BX51 microscope (Olympus). Quantitative analysis of the cross-sectional areas of individual muscle fibers (mean of 521 and 707 myofibers for diaphragm and TA, respectively) and the percentage of fibers containing central nuclei [an index of previous necrosis and subsequent regeneration (Karpati *et al*, 1988)] were determined using a commercial software package (Image-Pro Plus, Media Cybernetics, Silver Springs, MD). To evaluate the level of muscle infiltration by MPs, tissue sections were incubated with anti-F4/80 (Abcam) and quantified using color segregation-based image analysis (Image-Pro Plus). Fibrosis and necrosis were assessed on randomly selected transverse sections (6–7 per group) by staining with Gomori's modified trichrome (Stedman *et al*, 1991) and HRP-goat anti-mouse IgG polyclonal antibody (1:500 dilution, Promega) (Weller *et al*, 1990), respectively. The positively stained areas were quantified (ImageJ, Bethesda, MD) and reported to the total section area. The data were collected and analyzed in a blinded fashion.

### Hydroxyproline assay

As described previously (Stedman *et al*, 1991), muscles were homogenized in 0.5 mol/l glacial acetic acid, dried, and hydrolyzed in 6 N HCl. The samples were then processed using chloramine-T and Ehrlich's solutions, heated, and optical densities were read at 550 nm. Sample hydroxyproline content was referenced to a standard curve and expressed per mg of wet muscle weight. All chemicals were purchased from Sigma.

### Bone marrow-derived macrophage cultures

To isolate bone marrow cells, femur and tibiae were cut open at the epiphyses and the marrow flushed out using RPMI. The cells were washed with RPMI and counted using Trypan blue staining. Cells were plated onto 6-well plates at a frequency of 5 × 10^6^ cells/well and cultured for 7 days in complete RPMI supplemented with 10% fetal bovine serum (FBS) and 10% L929 conditioned medium (containing M-CSF). The adherent cells at the end of 7 days culture were used for mRNA quantification.

### Evaluation of force-generating capacity

The diaphragm was removed and placed into equilibrated (95% O_2_–5% CO_2_; pH 7.38) Krebs solution. After attaching the diaphragm strip to a force transducer/length servomotor system (model 300B; dual mode; Cambridge Technology, Watertown, MA), optimal length (*Lo*) was determined. The force–frequency relationship was measured by sequential supramaximal stimulation for 1 s at 10, 30, 50, 100, and 150 Hz, with 2 min between each stimulation train. A similar procedure was followed for the TA, except that the muscle was studied *in situ* during surgical anesthesia with ketamine (130 mg/kg) and xylazine (20 mg/kg). After securing the knee and ankle to a platform base, the skin of the lower hindlimb was opened to expose the TA. The distal tendon of the TA was then attached to the force transducer/length servomotor (model 305B dual mode; Cambridge Technology). After determining *Lo*, the TA was stimulated directly via an electrode placed on the belly of the muscle at 20, 50, 100, and 120 Hz. All muscle force data were acquired to computer at a sampling rate of 1,000 Hz for later analysis. After allowing a 10-min recovery period from the above measurements of isometric contractile properties, the diaphragm and TA muscles were further studied to evaluate the force drop after undergoing eccentric (lengthening) contractions; the protocol has been described in detail previously (Petrof *et al*, 1993; Dudley *et al*, 2006). Muscle force was normalized to cross-sectional area to determine specific force, which is expressed in Newtons/cm^2^.

### Pharmacological anti-CCR2 treatment

Mesenchymal stromal cells from CCL2^−/−^ mice were retrovirally transduced to express a CCR2-inhibiting ‘fusokine’ molecule, consisting of GM-CSF and truncated CCL2 (lacking the first five amino acids), also referred to in prior publications as GMME1 (Rafei *et al*, 2009, 2011). At 3 weeks of age in male mdx mice, approximately 5 million cells were admixed with a matrix substrate and implanted subcutaneously to form a fusokine-secreting organoid. For the control group, an identical organoid composed of non-secreting mesenchymal stromal cells was used. Secretion of the CCR2-inhibiting fusokine by the engineered mesenchymal stromal cells was verified using a standard ELISA for detection of CCL2 (eBioscience). Animals were assigned randomly to the treatment or control groups, and the data were collected and analyzed in a blinded fashion.

### Statistical analysis

All data are expressed as group mean values ± SE. Data were analyzed using commercial software (Minitab 17.1, Minitab Inc., State College, PA, USA; SPSS v11.5, IBM Corporation, Armonk, NY, USA). For each experiment, we evaluated the sample size according to an estimated standard deviation of samples based on pilot experiments and/or prior studies. Collected data were assessed for normality, and significant differences between groups were determined by *t*-test (if 2 groups), or by ANOVA with *post hoc* application of the Tukey test to adjust for multiple comparisons (if > 2 groups). Non-normally distributed data sets were first transformed using Box-Cox transformation before the statistical tests were applied. The level of significance was set at *P* < 0.05.

## References

[b1] Acharyya S, Villalta SA, Bakkar N, Bupha-Intr T, Janssen PM, Carathers M, Li ZW, Beg AA, Ghosh S, Sahenk Z (2007). Interplay of IKK/NF-kappaB signaling in macrophages and myofibers promotes muscle degeneration in Duchenne muscular dystrophy. J Clin Invest.

[b2] Ajami B, Bennett JL, Krieger C, McNagny KM, Rossi FM (2011). Infiltrating monocytes trigger EAE progression, but do not contribute to the resident microglia pool. Nat Neurosci.

[b3] Amalfitano A, Chamberlain JS (1996). The mdx-amplification-resistant mutation system assay, a simple and rapid polymerase chain reaction-based detection of the mdx allele. Muscle Nerve.

[b4] Arnold L, Henry A, Poron F, Baba-Amer Y, van Rooijen N, Plonquet A, Gherardi RK, Chazaud B (2007). Inflammatory monocytes recruited after skeletal muscle injury switch into antiinflammatory macrophages to support myogenesis. J Exp Med.

[b5] Auffray C, Fogg D, Garfa M, Elain G, Join-Lambert O, Kayal S, Sarnacki S, Cumano A, Lauvau G, Geissmann F (2007). Monitoring of blood vessels and tissues by a population of monocytes with patrolling behavior. Science.

[b6] Beastrom N, Lu H, Macke A, Canan BD, Johnson EK, Penton CM, Kaspar BK, Rodino-Klapac LR, Zhou L, Janssen PM (2011). Mdx( cv) mice manifest more severe muscle dysfunction and diaphragm force deficits than do mdx mice. Am J Pathol.

[b7] Bellinger AM, Reiken S, Carlson C, Mongillo M, Liu X, Rothman L, Matecki S, Lacampagne A, Marks AR (2009). Hypernitrosylated ryanodine receptor calcium release channels are leaky in dystrophic muscle. Nat Med.

[b8] Brigitte M, Schilte C, Plonquet A, Baba-Amer Y, Henri A, Charlier C, Tajbakhsh S, Albert M, Gherardi RK, Chrétien F (2010). Muscle resident macrophages control the immune cell reaction in a mouse model of notexin-induced myoinjury. Arthritis Rheum.

[b9] Burzyn D, Kuswanto W, Kolodin D, Shadrach JL, Cerletti M, Jang Y, Sefik E, Tan TG, Wagers AJ, Benoist C (2013). A special population of regulatory T cells potentiates muscle repair. Cell.

[b10] Chen YW, Nagaraju K, Bakay M, McIntyre O, Rawat R, Shi R, Hoffman EP (2005). Early onset of inflammation and later involvement of TGFbeta in Duchenne muscular dystrophy. Neurology.

[b11] Contreras-Shannon V, Ochoa O, Reyes-Reyna SM, Sun D, Michalek JE, Kuziel WA, McManus LM, Shireman PK (2007). Fat accumulation with altered inflammation and regeneration in skeletal muscle of CCR2-/- mice following ischemic injury. Am J Physiol Cell Physiol.

[b12] Côté CH, Bouchard P, van Rooijen N, Marsolais D, Duchesne E (2013). Monocyte depletion increases local proliferation of macrophage subsets after skeletal muscle injury. BMC Musculoskelet Disord.

[b13] Dudley RW, Danialou G, Govindaraju K, Lands L, Eidelman DE, Petrof BJ (2006). Sarcolemmal damage in dystrophin deficiency is modulated by synergistic interactions between mechanical and oxidative/nitrosative stresses. Am J Pathol.

[b14] Geissmann F, Jung S, Littman DR (2003). Blood monocytes consist of two principal subsets with distinct migratory properties. Immunity.

[b15] Gilbert J, Lekstrom-Himes J, Donaldson D, Lee Y, Hu M, Xu J, Wyant T, Davidson M, Group MS (2011). Effect of CC chemokine receptor 2 CCR2 blockade on serum C-reactive protein in individuals at atherosclerotic risk and with a single nucleotide polymorphism of the monocyte chemoattractant protein-1 promoter region. Am J Cardiol.

[b16] Hashimoto D, Chow A, Noizat C, Teo P, Beasley MB, Leboeuf M, Becker CD, See P, Price J, Lucas D (2013). Tissue-resident macrophages self-maintain locally throughout adult life with minimal contribution from circulating monocytes. Immunity.

[b17] Ingersoll MA, Platt AM, Potteaux S, Randolph GJ (2011). Monocyte trafficking in acute and chronic inflammation. Trends Immunol.

[b18] Izikson L, Klein RS, Charo IF, Weiner HL, Luster AD (2000). Resistance to experimental autoimmune encephalomyelitis in mice lacking the CC chemokine receptor (CCR)2. J Exp Med.

[b19] Jenkins SJ, Ruckerl D, Cook PC, Jones LH, Finkelman FD, van Rooijen N, MacDonald AS, Allen JE (2011). Local macrophage proliferation, rather than recruitment from the blood, is a signature of TH2 inflammation. Science.

[b20] Karpati G, Carpenter S, Prescott S (1988). Small-caliber skeletal muscle fibers do not suffer necrosis in mdx mouse dystrophy. Muscle Nerve.

[b21] Kim Y, Sung S, Kuziel WA, Feldman S, Fu SM, Rose CE (2001). Enhanced airway Th2 response after allergen challenge in mice deficient in CC chemokine receptor-2 (CCR2). J Immunol.

[b22] Lu H, Huang D, Saederup N, Charo IF, Ransohoff RM, Zhou L (2011). Macrophages recruited via CCR2 produce insulin-like growth factor-1 to repair acute skeletal muscle injury. FASEB J.

[b23] Lumeng CN, Bodzin JL, Saltiel AR (2007). Obesity induces a phenotypic switch in adipose tissue macrophage polarization. J Clin Invest.

[b24] Mantovani A, Sica A, Sozzani S, Allavena P, Vecchi A, Locati M (2004). The chemokine system in diverse forms of macrophage activation and polarization. Trends Immunol.

[b25] Manzur AY, Kuntzer T, Pike M, Swan A (2008). Glucocorticoid corticosteroids for Duchenne muscular dystrophy. Cochrane Database Syst Rev.

[b26] Martinez CO, McHale MJ, Wells JT, Ochoa O, Michalek JE, McManus LM, Shireman PK (2010). Regulation of skeletal muscle regeneration by CCR2-activating chemokines is directly related to macrophage recruitment. Am J Physiol Regul Integr Comp Physiol.

[b27] Martinez FO, Helming L, Gordon S (2009). Alternative activation of macrophages: an immunologic functional perspective. Annu Rev Immunol.

[b28] Messina S, Bitto A, Aguennouz M, Minutoli L, Monici MC, Altavilla D, Squadrito F, Vita G (2006). Nuclear factor kappa-B blockade reduces skeletal muscle degeneration and enhances muscle function in Mdx mice. Exp Neurol.

[b29] Mildner A, Mack M, Schmidt H, Brück W, Djukic M, Zabel MD, Hille A, Priller J, Prinz M (2009). CCR2+Ly-6Chi monocytes are crucial for the effector phase of autoimmunity in the central nervous system. Brain.

[b30] Moore BB, Kolodsick JE, Thannickal VJ, Cooke K, Moore TA, Hogaboam C, Wilke CA, Toews GB (2005). CCR2-mediated recruitment of fibrocytes to the alveolar space after fibrotic injury. Am J Pathol.

[b31] Mosser DM, Edwards JP (2008). Exploring the full spectrum of macrophage activation. Nat Rev Immunol.

[b32] Nahrendorf M, Swirski FK, Aikawa E, Stangenberg L, Wurdinger T, Figueiredo JL, Libby P, Weissleder R, Pittet MJ (2007). The healing myocardium sequentially mobilizes two monocyte subsets with divergent and complementary functions. J Exp Med.

[b33] Nelson CA, Hunter RB, Quigley LA, Girgenrath S, Weber WD, McCullough JA, Dinardo CJ, Keefe KA, Ceci L, Clayton NP (2011). Inhibiting TGF-beta activity improves respiratory function in mdx mice. Am J Pathol.

[b34] Nio Y, Yamauchi T, Iwabu M, Okada-Iwabu M, Funata M, Yamaguchi M, Ueki K, Kadowaki T (2012). Monocyte chemoattractant protein-1 (MCP-1) deficiency enhances alternatively activated M2 macrophages and ameliorates insulin resistance and fatty liver in lipoatrophic diabetic A-ZIP transgenic mice. Diabetologia.

[b35] Onozuka I, Kakinuma S, Kamiya A, Miyoshi M, Sakamoto N, Kiyohashi K, Watanabe T, Funaoka Y, Ueyama M, Nakagawa M (2011). Cholestatic liver fibrosis and toxin-induced fibrosis are exacerbated in matrix metalloproteinase-2 deficient mice. Biochem Biophys Res Commun.

[b36] Pesce JT, Ramalingam TR, Mentink-Kane MM, Wilson MS, El Kasmi KC, Smith AM, Thompson RW, Cheever AW, Murray PJ, Wynn TA (2009). Arginase-1-expressing macrophages suppress Th2 cytokine-driven inflammation and fibrosis. PLoS Pathog.

[b37] Petrof BJ, Shrager JB, Stedman HH, Kelly AM, Sweeney HL (1993). Dystrophin protects the sarcolemma from stresses developed during muscle contraction. Proc Natl Acad Sci U S A.

[b38] Petrof BJ (2002). Molecular pathophysiology of myofiber injury in deficiencies of the dystrophin-glycoprotein complex. Am J Phys Med Rehabil.

[b39] Piers AT, Lavin T, Radley-Crabb HG, Bakker AJ, Grounds MD, Pinniger GJ (2011). Blockade of TNF in vivo using cV1q antibody reduces contractile dysfunction of skeletal muscle in response to eccentric exercise in dystrophic mdx and normal mice. Neuromuscul Disord.

[b40] Porter JD, Khanna S, Kaminski HJ, Rao JS, Merriam AP, Richmonds CR, Leahy P, Li J, Guo W, Andrade FH (2002). A chronic inflammatory response dominates the skeletal muscle molecular signature in dystrophin-deficient mdx mice. Hum Mol Genet.

[b41] Qian BZ, Li J, Zhang H, Kitamura T, Zhang J, Campion LR, Kaiser EA, Snyder LA, Pollard JW (2011). CCL2 recruits inflammatory monocytes to facilitate breast-tumour metastasis. Nature.

[b42] Radley HG, Davies MJ, Grounds MD (2008). Reduced muscle necrosis and long-term benefits in dystrophic mdx mice after cV1q (blockade of TNF) treatment. Neuromuscul Disord.

[b43] Rafei M, Berchiche YA, Birman E, Boivin MN, Young YK, Wu JH, Heveker N, Galipeau J (2009). An engineered GM-CSF-CCL2 fusokine is a potent inhibitor of CCR2-driven inflammation as demonstrated in a murine model of inflammatory arthritis. J Immunol.

[b44] Rafei M, Deng J, Boivin MN, Williams P, Matulis SM, Yuan S, Birman E, Forner K, Yuan L, Castellino C (2011). A MCP1 fusokine with CCR2-specific tumoricidal activity. Mol Cancer.

[b45] Robbins CS, Hilgendorf I, Weber GF, Theurl I, Iwamoto Y, Figueiredo JL, Gorbatov R, Sukhova GK, Gerhardt LM, Smyth D (2013). Local proliferation dominates lesional macrophage accumulation in atherosclerosis. Nat Med.

[b46] Saclier M, Cuvellier S, Magnan M, Mounier R, Chazaud B (2013). Monocyte/macrophage interactions with myogenic precursor cells during skeletal muscle regeneration. FEBS J.

[b47] Schulz C, Gomez Perdiguero E, Chorro L, Szabo-Rogers H, Cagnard N, Kierdorf K, Prinz M, Wu B, Jacobsen SE, Pollard JW (2012). A lineage of myeloid cells independent of Myb and hematopoietic stem cells. Science.

[b48] Serbina NV, Pamer EG (2006). Monocyte emigration from bone marrow during bacterial infection requires signals mediated by chemokine receptor CCR2. Nat Immunol.

[b49] Shi C, Pamer EG (2011). Monocyte recruitment during infection and inflammation. Nat Rev Immunol.

[b50] Shireman PK, Contreras-Shannon V, Ochoa O, Karia BP, Michalek JE, McManus LM (2007). MCP-1 deficiency causes altered inflammation with impaired skeletal muscle regeneration. J Leukoc Biol.

[b51] Sica A, Mantovani A (2012). Macrophage plasticity and polarization: in vivo veritas. J Clin Invest.

[b52] Spencer MJ, Montecino-Rodriguez E, Dorshkind K, Tidball JG (2001). Helper (CD4(+)) and cytotoxic (CD8(+)) T cells promote the pathology of dystrophin-deficient muscle. Clin Immunol.

[b53] Stedman HH, Sweeney HL, Shrager JB, Maguire HC, Panettieri RA, Petrof B, Narusawa M, Leferovich JM, Sladky JT, Kelly AM (1991). The mdx mouse diaphragm reproduces the degenerative changes of Duchenne muscular dystrophy. Nature.

[b54] Sun D, Martinez CO, Ochoa O, Ruiz-Willhite L, Bonilla JR, Centonze VE, Waite LL, Michalek JE, McManus LM, Shireman PK (2009). Bone marrow-derived cell regulation of skeletal muscle regeneration. FASEB J.

[b55] Swirski FK, Nahrendorf M, Etzrodt M, Wildgruber M, Cortez-Retamozo V, Panizzi P, Figueiredo JL, Kohler RH, Chudnovskiy A, Waterman P (2009). Identification of splenic reservoir monocytes and their deployment to inflammatory sites. Science.

[b56] Traynor TR, Kuziel WA, Toews GB, Huffnagle GB (2000). CCR2 expression determines T1 versus T2 polarization during pulmonary *Cryptococcus neoformans* infection. J Immunol.

[b57] Tsou CL, Peters W, Si Y, Slaymaker S, Aslanian AM, Weisberg SP, Mack M, Charo IF (2007). Critical roles for CCR2 and MCP-3 in monocyte mobilization from bone marrow and recruitment to inflammatory sites. J Clin Invest.

[b58] Vetrone SA, Montecino-Rodriguez E, Kudryashova E, Kramerova I, Hoffman EP, Liu SD, Miceli MC, Spencer MJ (2009). Osteopontin promotes fibrosis in dystrophic mouse muscle by modulating immune cell subsets and intramuscular TGF-beta. J Clin Invest.

[b59] Vidal B, Serrano AL, Tjwa M, Suelves M, Ardite E, De Mori R, Baeza-Raja B, Martínez de Lagrán M, Lafuste P, Ruiz-Bonilla V (2008). Fibrinogen drives dystrophic muscle fibrosis via a TGFbeta/alternative macrophage activation pathway. Genes Dev.

[b60] Villalta SA, Nguyen HX, Deng B, Gotoh T, Tidball JG (2009). Shifts in macrophage phenotypes and macrophage competition for arginine metabolism affect the severity of muscle pathology in muscular dystrophy. Hum Mol Genet.

[b61] Warren GL, Hulderman T, Mishra D, Gao X, Millecchia L, O'Farrell L, Kuziel WA, Simeonova PP (2005). Chemokine receptor CCR2 involvement in skeletal muscle regeneration. FASEB J.

[b62] Wehling M, Spencer MJ, Tidball JG (2001). A nitric oxide synthase transgene ameliorates muscular dystrophy in mdx mice. J Cell Biol.

[b63] Weller B, Karpati G, Carpenter S (1990). Dystrophin-deficient mdx muscle fibers are preferentially vulnerable to necrosis induced by experimental lengthening contractions. J Neurol Sci.

[b64] Whitehead NP, Pham C, Gervasio OL, Allen DG (2008). N-Acetylcysteine ameliorates skeletal muscle pathophysiology in mdx mice. J Physiol.

[b65] Yahiaoui L, Gvozdic D, Danialou G, Mack M, Petrof BJ (2008). CC family chemokines directly regulate myoblast responses to skeletal muscle injury. J Physiol.

[b66] Yona S, Kim KW, Wolf Y, Mildner A, Varol D, Breker M, Strauss-Ayali D, Viukov S, Guilliams M, Misharin A (2013). Fate mapping reveals origins and dynamics of monocytes and tissue macrophages under homeostasis. Immunity.

