# Supplementary material for: Inflammatory monocytes promote progression of Duchenne muscular dystrophy and can be therapeutically targeted via CCR2
Source: EMBO Mol Med. 2014 Oct 13;6(11):1476–92. doi: 10.15252/emmm.201403967 (PMC4237472; doi:10.15252/emmm.201403967)
Supplement: Supplementary file 1 [file emmm0006-1476-sd1.pdf]

EMBO Molecular Medicine

Inflammatory monocytes promote progression of Duchenne muscular dystrophy and can be therapeutically targeted via CCR2

Kamalika Mojumdar, Feng Liang, Christian Giordano, Christian Lemaire, Gawiyou Danialou, Tatsuma Okazaki, Johanne Bourdon, Moutih Rafei, Jacques Galipeau, Maziar Divangahi, Basil J. Petrof

Corresponding author: Basil Petrof, McGill University, Meakins-Christie Laboratories

Review timeline:

Submission date:	17 February 2014
Editorial Decision:	10 April 2014
Revision received:	10 July 2014
Editorial Decision:	13 August 2014
Appeal:	25 August 2014
Editorial Decision:	03 September 2014
Additional Author Correspondence:	04 September 2014
Revision received:	16 September 2014
Accepted:	18 September 2014

Transaction Report:

(Note: With the exception of the correction of typographical or spelling errors that could be a source of ambiguity, letters and reports are not edited. The original formatting of letters and referee reports may not be reflected in this compilation.)

Editors: Céline Carret / Roberto Buccione

1st Editorial Decision 10 April 2014

Thank you for the submission of your manuscript to EMBO Molecular Medicine. I am very sorry for the long time it took us to get back to you. Unfortunately, a referee dropped out at the last moment so we had to ask an editorial board adviser to help reach the best possible decision. We have now heard back from the two referees whom we asked to evaluate your manuscript and from this advisor.

You will see that they all find the topic of your manuscript interesting but they feel that the data need to be strengthened and the main message clarified. As we find the suggestions constructive and self explanatory enough, we would encourage you to address these criticisms in full, experimentally when requested. Should you be able to respond to all concerns satisfactorily, we would be happy to consider a revised manuscript.

Please note that it is EMBO Molecular Medicine policy to allow a single round of revision in order to avoid the delayed publication of research findings. Consequently, acceptance or rejection of the manuscript will depend on the completeness of your responses included in the next version of the manuscript.

EMBO Molecular Medicine has a "scooping protection" policy, whereby similar findings that are

published by others during review or revision are not a criterion for rejection. Should you decide to submit a revised version, I do ask that you get in touch after three months if you have not completed it, to update us on the status.

Please also contact us as soon as possible if similar work is published elsewhere. If other work is published we may not be able to extend the revision period beyond three months.

I look forward to seeing a revised form of your manuscript as soon as possible.

***** Reviewer's comments *****

Referee #1 (Comments on Novelty/Model System):

The model used, mdx diaphragm is totally adequate to study Duchenne Dystrophy.
The role of CCR2/CCL2 axis, although extensively studied in normal muscle, has not been investigated in muscle diseases.
The results showing that a competitor/inhibitor (endogenously delivered) of the CCR2/CCL2 axis improves muscle phenotype leads to consider the development of a systemic treatment targeting circulating monocytes in this disease.

Referee #1 (Remarks):

As a whole, the study by Liang et al is well conducted, clear, the iconography is very good and the results are correctly interpreted. Few comments are given and some additional experiments are suggested to reinforce the message of the work, and to make the story complete.

-Results - page 7, Figure 2. Authors show a reduction of MP number in mdxCCR2 KO as compared with mdx at 6 weeks. Looking at the analyses in Fig2, it is not clear whether the reduction from 72 to 65% (thus about 10% reduction) observed in FigA can account for the reduction from 11000 to 5000 macrophages (thus more than 50% reduction) in Fig.B. Does it mean that the total CD45+ cells is also reduced (meaning that CCR2 deficiency also affects other CD45+ cells)? Please clarify.
- Results - page 8, Figure 3. The analysis in blood presented in Fig3 should be also performed in WT mouse, as it has been done for the muscle in Fig2. This would give some information on the homeostasis of monocytes in normal versus diseased animals.
- Results - page 8, Figure 4. What about the macrophages in skeletal muscle in splenectomized animals? Same experiments as in Fig2A should be provided, both in % CD45+ cells and in absolute number, to formally assess the absence of a role of spleen-derived monocytes in DMD.
- Results - page 9, Figure 5. There is here a conceptual and technical concern. The statement that iNOS+ CD206- and iNOS-CD206+ are M1 and M2 macrophages, respectively, is not correct. First, several studies have shown the huge heterogeneity of the expression of M1 and M2 markers in macrophage populations, notably in muscle (Mounier et al., 2013). Both types of macrophages are capable of synthesising both types of markers. Second, when looking at the cytometry analysis, one can see 2 discrete populations in WT muscle while in mdx only one cloud of macrophages is observed. This suggests that the phenotype of the macrophagic population is completely different. The shift on the right/left axis certainly reflects a change in the expression of CD206 but cannot make the cells fall in a "M1" or "M2" category. Without the use of more markers, cells should not be tagged as M1 or M2. Finally, it does not seem that iNOS expression was altered in mdxCCR2 as compared with mdx. Is that correct? If yes, a M2/M1 ratio is not informative. I suggest that either the authors use a battery of markers to prove the existence of M1 and M2 populations in mdx, or they present the results as a shift of FMI in CD206 (and iNOS) showing a "recovery" of the phenotype of macrophages in mdxCCR2. The final message is the same, but the statement is in full adequation with the results, since the populations of macrophages are still not characterized in mdx.
- Results - page 10. The reason why measurements have been done in TA should be introduced and explained (since the whole study has been done in diaphragm until this point).
- Results - page 11. How the authors explain an improvement of physiological parameters at 6 weeks without measurable histological improvement?
- Remove Fig10.

- Is there an alteration of the LY6Cneg pool of monocytes in mdx and mdxCCR2 circulation? It looks like in Fig3A that this population is increased in mdx as compared with mdxCCR2- (again the WT is missing here)
- Finally, what about the resident cells in the muscle? As proposed by the authors in the discussion page 17, they may easily investigate this issue. Fig2E shows a ratio between Ly6Chi and Ly6Clo. What is the absolute number of each population in WT, mdx and mdxCCR2KO? If there is an increased of Ly6Cneg in one or the other context, it would be nice to analyse the cell cycle of these cells, known to self-renew (either by BrdU by FACS, or PCNA or ki67 after cytospining of the sorted populations). This would provide a complete picture of all populations in blood and muscle in both normal, mdx and mdxCCR2 KO animals.
- Discussion page 15, line 6, remove/dampen the "increased relative proportion of M2"

Referee #2 (Remarks):

This is a well-written paper from Liang et al evaluating the role of CCR2 on the disease phenotype in mdx mice. Authors showed that CCR2 and its ligands were increased in mdx mice at 6 and 12-weeks age. CCR2 deficient mdx mice showed decreased central nucleated fibers, increased mean cross sectional area, degreased fibrosis and increased force generation. Likewise they also suggested that pharmacological blocking of CCR2 results in functional improvements in mdx mice. There are several major concerns

1. What is the genetic background of the mdx-CCR2-/- mice? If they were backcrossed it would be important to include that information in the methods along with the PCR genotyping protocols used to screen the mice.
2. CCR2 and CCR2 ligand expression data presented in Figure 1 indicate that the expression of these ligands increases in 12-week old mice that generally show less inflammatory response in comparison to 6-week old mice. How do you reconcile almost 3 fold lower macrophages and 2-fold increase in CCL2 and CCL7 expression in diaphragm at 12-weeks age?
3. Interpretation of CD11b expression data may not be entirely accurate because there is clearly an intermediate population of CD11b positive cells in 12-week old mdx-CCR2-/- muscle (Figure 2E). CD11b positive intermediate population appears to be significantly increased in 12-week old mdx-CCR2-/-mice. It would be helpful to know the nature of these cells in 12-week old mdx-CCR2-/- muscle.
4. It would be useful to know the CCR2 expression on Ly6C high and low populations to better understand the origin of these cells.
5. Is there a difference in the %CNF in mdx-CCR2-/- mice at 6 and 12 weeks age?
6. Authors indicate that average cross sectional are of the regenerating fibers (Fig6D-E) was larger in the mdx-CCR2-/- group at 12 weeks suggesting more effective regeneration. It would be helpful to know how they calculated cross sectional area of the regenerating fibers? Is there evidence that demonstrates bigger cross sectional area means more effective regeneration?
7. It would be highly useful to provide experimental evidence that demonstrates the mechanism by which CCR2 deficiency results in fibrosis reduction in mdx mice.
8. Authors claim that CCR2 ablation reduces cycles of necrosis and promotes more effective muscle repair leading to decreased fibrosis. There is no data that demonstrates reduced necrosis or effective muscle repair in mdx-CCR2 mice.
9. It would be helpful to provide information on the sex, whether animal were randomized before anti-CCR2 treatment. Was the data acquired in a blinded fashion?
10. It would be helpful to know that pharmacological anti-CCR2 treatment actually blocked the CCR2. If so what is the effect of treatment on MO/MQ infiltrates in the skeletal muscle? What is the effect of treatment on fibrosis?
11. Specific force (N/cm2) data (Figure 9F) indicates that controls generates <10N/cm2 at all frequencies. On the other hand mdx mice general show >10N/cm2 after 50Hz (Figure 8 A & B). Please clarify?

1st Revision - authors' response 10 July 2014

Referee #1

Comments on Novelty/Model System:

The model used, mdx diaphragm is totally adequate to study Duchenne Dystrophy.

The role of CCR2/CCL2 axis, although extensively studied in normal muscle, has not been investigated in muscle diseases.

The results showing that a competitor/inhibitor (endogenously delivered) of the CCR2/CCL2 axis improves muscle phenotype leads to consider the development of a systemic treatment targeting circulating monocytes in this disease.

We thank the reviewer for these encouraging remarks and the very constructive suggestions which have served to improve the quality of the paper. Below are specific point-by-point responses to the reviewer's comments.

Remarks:

As a whole, the study by Liang et al is well conducted, clear, the iconography is very good and the results are correctly interpreted. Few comments are given and some additional experiments are suggested to reinforce the message of the work, and to make the story complete.

1. *Results - page 7, Figure 2. Authors show a reduction of MP number in mdxCCR2 KO as compared with mdx at 6 weeks. Looking at the analyses in Fig2, it is not clear whether the reduction from 72 to 65% (thus about 10% reduction) observed in Fig A can account for the reduction from 11000 to 5000 macrophages (thus more than 50% reduction) in Fig. B. Does it mean that the total CD45+ cells is also reduced (meaning that CCR2 deficiency also affects other CD45+ cells)? Please clarify.*

Response: The reviewer is correct. The reduction in absolute macrophage numbers in mdx versus mdx-CCR2-/- at 6 weeks is partially accounted for by a decrease in total CD45+ cells. In this regard, there was a group mean 57% reduction in total macrophages as compared with a 40% reduction in total CD45+ cells. As discussed in the original manuscript, we do not exclude the possibility that part of the benefits of CCR2 deficiency are related to effects on other cell types besides monocytes/macrophages. This could include other leukocyte populations such as T cells as well as non-leukocyte cell types such as circulating fibrocytes (Moore et al, 2005), which have been described as being CD45+ in previous publications. These are areas that we are now in the process of investigating further, but these studies each require extensive experimentation. We note these possibilities in the revised discussion section (page 19, paragraph 1) and also provide the data on absolute numbers of CD45+ cells (page 7, paragraph 1).

2. *Results - page 8, Figure 3. The analysis in blood presented in Fig3 should be also performed in WT mouse, as it has been done for the muscle in Fig2. This would give some information on the homeostasis of monocytes in normal versus diseased animals.*

Response: We have now included WT data for blood monocytes in the figure as requested by the reviewer (new Fig. 4 in revised manuscript).

3. *Results - page 8, Figure 4. What about the macrophages in skeletal muscle in splenectomised animals? Same experiments as in Fig2A should be provided, both in % CD45+ cells and in*

absolute number, to formally assess the absence of a role of spleen-derived monocytes in DMD.

Response: As suggested by the reviewer, we have now performed an entirely new set of experiments in splenectomised and sham-control animals, in order to assess the role of spleen-derived monocytes on macrophage infiltration in the diaphragm of mdx animals. As now shown in the revised manuscript (new Figs. 5A and B), splenectomy did not significantly alter the macrophage proportion (% of CD45+ cells) or the absolute numbers of diaphragm-infiltrating macrophages in mdx mice. Therefore, these data show that splenectomy failed to modify either the macrophage content or the histological/physiological functional features of muscle pathology in these animals.

4. *Results - page 9, Figure 5. There is here a conceptual and technical concern. The statement that iNOS+ CD206- and iNOS-CD206+ are M1 and M2 macrophages, respectively, is not correct. First, several studies have shown the huge heterogeneity of the expression of M1 and M2 markers in macrophage populations, notably in muscle (Mounier et al., 2013). Both types of macrophages are capable of synthesising both types of markers. Second, when looking at the cytometry analysis, one can see 2 discrete populations in WT muscle while in mdx only one cloud of macrophages is observed. This suggests that the phenotype of the macrophagic population is completely different. The shift on the right/left axis certainly reflects a change in the expression of CD206 but cannot make the cells fall in a "M1" or "M2" category. Without the use of more markers, cells should not be tagged as M1 or M2. Finally, it does not seem that iNOS expression was altered in mdxCCR2 as compared with mdx. Is that correct? If yes, a M2/M1 ratio is not informative. I suggest that either the authors use a battery of markers to prove the existence of M1 and M2 populations in mdx, or they present the results as a shift of FMI in CD206 (and iNOS) showing a "recovery" of the phenotype of macrophages in mdxCCR2. The final message is the same, but the statement is in full adequation with the results, since the populations of macrophages are still not characterized in mdx.*

Response: Thank you for these comments and helpful suggestions. As indicated, the two discrete populations we observed in the WT group (based on the classical M1 and M2 markers, iNOS and CD206) initially led us to quantify these populations and categorize them as M1 and M2 in the 3 mouse strains. However, we fully agree with the reviewer that macrophage phenotype is highly heterogeneous and our approach does not fully capture the complexity of muscle macrophage characteristics, particularly in the dystrophic animals. Accordingly, we no longer employ the nomenclature of “M1” and “M2”, and have eliminated the M1/M2 ratio graph as requested. In addition, as suggested by the reviewer, we have now quantified the iNOS and CD206 MFIs on the muscle macrophages from WT, mdx and mdx-CCR2-/- mice. As the reviewer has correctly observed, iNOS MFI values did not differ between the mdx and mdx-CCR2-/- groups. However, one does indeed see a significant shift toward greater CD206 MFI values in mdx-CCR2-/- macrophages, thus indicating a recovery of this parameter to normal WT values in the mdx mice which are CCR2 deficient. These changes and new data are provided in Fig. 6 of the revised manuscript, and appropriate modifications have also been made to the relevant portions of the discussion section (page 10, middle paragraph).

5. *Results - page 10. The reason why measurements have been done in TA should be introduced and explained (since the whole study has been done in diaphragm until this point).*

Response: The primary focus of this paper is indeed the mdx mouse diaphragm due to its greater resemblance to the human DMD phenotype (Stedman et al, 1991). Because some investigators in the field also use the limb muscle (and particularly the TA) to test various therapeutic approaches in mdx mice, we had also included more limited outcome data in this muscle. However, in view of the reviewer’s comments and to improve the focus as well as reduce the length of the manuscript (since we have added substantial new data), the TA findings have been removed from the main body of the paper but can still be found in the online supplement. We have also added a brief rationale for studying the TA in the revised methods section (page 20, paragraph 1).

6. *Results - page 11. How the authors explain an improvement of physiological parameters at 6 weeks without measurable histological improvement?*

Response: The reviewer raises an interesting question which is currently poorly understood in the field: What exactly causes the loss of specific force in dystrophic muscles? It is clear that the loss of force is not only related to the amount of necrosis or fibrosis observed histologically in the muscle tissue, as these parameters do not correlate well with measurements of specific force (i.e., force normalized to muscle cross-sectional area) in individual muscles. Therefore, it is likely that a major component of the muscle weakness, particularly at early stages of the disease, is related to subcellular alterations of contractile mechanisms which are not visible histologically. This would include the adverse effects of force-inhibiting mediators such as inflammatory cytokines (Piers et al 2011), oxidative stress (Whitehead et al, 2008), and abnormal calcium homeostasis (Bellinger et al, 2009). Such abnormalities leading to contractile dysfunction have all been described in dystrophic muscles. Accordingly, we speculate that the significant reduction in total macrophage numbers in the 6-week mdx-CCR2-/-, together with the fact that these macrophages exhibited higher expression of CD206 consistent with a less inflammatory phenotype, most likely reduced exposure of muscle fibres to force-inhibiting mediators which are not reflected by gross histological changes. This issue is now commented upon in the revised manuscript (page 16, paragraph 1).

7. *Remove Fig 10.*

Response: We have removed the figure as suggested.

8. *Is there an alteration of the LY6Cneg pool of monocytes in mdx and mdxCCR2 circulation? It looks like in Fig3A that this population is increased in mdx as compared with mdxCCR2- (again the WT is missing here)*

Response: The group mean values for the Ly6C-neg monocyte pool were not significantly different between WT, mdx and mdx-CCR2-/- groups (p=0.21). However, we agree that the flow cytometry plot shown for mdx and mdx-CCR2-/- blood monocytes in the original figure may give this erroneous impression. Accordingly, we have now selected representative mdx plots which more appropriately reflect the group mean data and have also added the WT group as suggested (see Fig. 4 of the revised manuscript).

9. *Finally, what about the resident cells in the muscle? As proposed by the authors in the discussion page 17, they may easily investigate this issue. Fig2E shows a ratio between Ly6Chi and Ly6Clo. What is the absolute number of each population in WT, mdx and mdxCCR2KO? If there is an increased of Ly6Cneg in one or the other context, it would be nice to analyse the cell cycle of these cells, known to self-renew (either by BrdU by FACS, or PCNA or ki67 after cytospining of the sorted populations). This would provide a complete picture of all populations in blood and muscle in both normal, mdx and mdxCCR2 KO animals.*

Response: As requested, we have performed new experiments using Ki67 to characterize the proliferation status of the recruited and resident macrophage populations. These new data indicate that in mdx-CCR2-/- mice, there is significantly higher local proliferation of both the monocyte recruitment-derived macrophages (at 6 and 12 wks) and the resident macrophage population (at 12 weeks), relative to WT and mdx mice. We interpret these findings as reflecting an adaptive response to compensate for the reduced monocyte-derived macrophage recruitment to muscle in mdx-CCR2-/- mice. This observation is consistent with a recent report that clodronate-treated rats (depleted of monocytes) and then subjected to bupivacaine-induced injury, also showed an increased level of macrophage proliferation within the affected muscles (Côté et al, 2013). These issues are now further commented upon in the revised discussion section (bottom of page 17 and top of page 18). The data on macrophage proliferation are provided in an entirely new Fig. 3, together with the absolute numbers of recruited and resident macrophages in each mouse strain as requested by the

reviewer.

10. Discussion page 15, line 6, remove/dampen the "increased relative proportion of M2"

Response: We have made the requested change to the discussion section of the revised manuscript.

References Cited

Bellinger AM, Reiken S, Carlson C, Mongillo M, Liu X, Rothman L, Matecki S, Lacampagne A, Marks AR (2009) Hypernitrosylated ryanodine receptor calcium release channels are leaky in dystrophic muscle. *Nat Med* **15:** 325-330

Côté CH, Bouchard P, van Rooijen N, Marsolais D, Duchesne E (2013) Monocyte depletion increases local proliferation of macrophage subsets after skeletal muscle injury. *BMC Musculoskelet Disord* **14:** 359

Moore BB, Kolodsick JE, Thannickal VJ, Cooke K, Moore TA, Hogaboam C, Wilke CA, Toews GB (2005) CCR2-mediated recruitment of fibrocytes to the alveolar space after fibrotic injury. *Am J Pathol* **166:** 675-684

Piers AT, Lavin T, Radley-Crabb HG, Bakker AJ, Grounds MD, Pinniger GJ (2011) Blockade of TNF in vivo using cV1q antibody reduces contractile dysfunction of skeletal muscle in response to eccentric exercise in dystrophic mdx and normal mice. *Neuromuscul Disord* **21:** 132-141

Stedman HH, Sweeney HL, Shrager JB, Maguire HC, Panettieri RA, Petrof B, Narusawa M, Leferovich JM, Sladky JT, Kelly AM (1991) The mdx mouse diaphragm reproduces the degenerative changes of Duchenne muscular dystrophy. *Nature* **352:** 536-539

Whitehead NP, Pham C, Gervasio OL, Allen DG (2008) N-Acetylcysteine ameliorates skeletal muscle pathophysiology in mdx mice. *J Physiol* **586:** 2003-2014

Referee #2

Remarks:

This is a well-written paper from Liang et al evaluating the role of CCR2 on the disease phenotype in mdx mice. Authors showed that CCR2 and its ligands were increased in mdx mice at 6 and 12-weeks age. CCR2 deficient mdx mice showed decreased central nucleated fibres, increased mean cross sectional area, decreased fibrosis and increased force generation. Likewise they also suggested that pharmacological blocking of CCR2 results in functional improvements in mdx mice. There are several major concerns

1. *What is the genetic background of the mdx-CCR2-/- mice? If they were backcrossed it would be important to include that information in the methods along with the PCR genotyping protocols used to screen the mice.*

Response: The genetic background information and PCR genotyping protocol and primer sequences used to screen the mdx-CCR2-/- mice are now included in the methods section (page 20, paragraph 1).

2. *CCR2 and CCR2 ligand expression data presented in Figure 1 indicate that the expression of these ligands increases in 12-week old mice that generally show less inflammatory*

© EMBO

response in comparison to 6-week old mice. How do you reconcile almost 3 fold lower macrophages and 2-fold increase in CCL2 and CCL7 expression in diaphragm at 12-weeks age?

Response: We agree with the reviewer that this is an intriguing observation and we envision that this potentially can be explained by at least three mechanisms. First, it is possible that the increased magnitude and/or chronicity of exposure to CCL2/CCL7 in 12-week-old mdx mice resulted in some degree of CCR2 receptor desensitization (Aragay et al, 1998). Second, increased chemokine decoy receptor expression (Graham, 2009) may have led to greater scavenging of CCR2 ligands, which then reduced their biological impact upon monocyte/macrophage function. Third, CCR2 ligands are certainly not the only mediators capable of attracting monocytes to damaged tissues, and the importance of other chemotactic mediators relative to CCR2 ligands may vary according to age or disease stage in mdx mice. Additional detailed studies would be required to distinguish among these different possibilities.

3. *Interpretation of CD11b expression data may not be entirely accurate because there is clearly an intermediate population of CD11b positive cells in 12-week old mdx-CCR2-/- muscle (Figure 2E). CD11b positive intermediate population appears to be significantly increased in 12-week old mdx-CCR2-/-mice. It would be helpful to know the nature of these cells in 12-week old mdx-CCR2-/- muscle.*

Response: We agree that a population of macrophages with a more intermediate level of CD11b staining does appear to be present in the mdx-CCR2-/- group at 12 weeks. As the other reviewer recommended, we have performed new experiments to evaluate the proliferation status of macrophages within the diaphragm using Ki67. These experiments revealed that macrophages with positive Ki67 staining sometimes demonstrate a more intermediate CD11b expression level, and the highest percentage of such proliferating macrophages is found in 12-week-old mdx-CCR2-/- mice (see Fig. 3 of revised manuscript). Therefore, we believe that these proliferating macrophages may explain the more prominent CD11b-intermediate population in this group.

4. *It would be useful to know the CCR2 expression on Ly6C high and low populations to better understand the origin of these cells.*

Response: As suggested by the reviewer, we have analysed expression of CCR2 on Ly6C-high and Ly6C-low monocytes. Ly6C-high monocytes were almost exclusively CCR2-positive in WT and mdx mice, whereas Ly6C-low monocytes were largely CCR2-negative. These data are now presented in supplementary Fig. S2.

5. *Is there a difference in the %CNF in mdx-CCR2-/- mice at 6 and 12 weeks age?*

Response: There was no significant difference in the percentage of centrally nucleated fibres between the 6 and 12-week-old mdx-CCR2-/- groups ($p = 0.26$).

6. *Authors indicate that average cross sectional area of the regenerating fibres (Fig6D-E) was larger in the mdx-CCR2-/- group at 12 weeks suggesting more effective regeneration. It would be helpful to know how they calculated cross sectional area of the regenerating fibres? Is there evidence that demonstrates bigger cross sectional area means more effective regeneration?*

Response: The regenerated fibres in dystrophic muscle were defined by the presence of centrally located nuclei (Karpati et al, 1988). After capturing images of randomly selected microscopic fields to computer, quantitative analysis of the cross-sectional area of these regenerated fibres was determined by tracing the borders each individual centrally nucleated fibre, using a commercial

software package (Image-Pro Plus, Media Cybernetics, Silver Springs, MD). In view of the reviewer's comment, we have replaced the term "more effective regeneration" (which was based on the larger cross-sectional area of these regenerated centrally nucleated fibres in the mdx-CCR2-/- group) with "more effective reconstitution of fibber size" in order to be more accurate on this point (page 11, paragraph 1).

7. *It would be highly useful to provide experimental evidence that demonstrates the mechanism by which CCR2 deficiency results in fibrosis reduction in mdx mice.*

Response: This is an interesting but also rather complex issue. Although we do not have the definitive answer to the question, we have begun to address it by performing experiments to determine whether macrophages derived from mdx and mdx-CCR2-/- mice have intrinsic differences in their expression of fibrosis mediators. These experiments have revealed that mdx-CCR2-/- macrophages express lower levels of osteopontin (SPP-1) and higher levels of MMP-2 and MMP-9; this pattern of gene expression is consistent with a less fibrogenic phenotype (Cabrera et al, 2007; Onozuka et al, 2011; Radbill et al, 2011; Vetrone et al, 2009). Therefore, these new data do provide insight into the reviewer's question and are now shown in supplemental Fig. S6. However, we recognize that there are many other possible mechanisms, and we consider that this topic would need to form the basis for an entirely separate paper to comprehensively address the question.

8. *Authors claim that CCR2 ablation reduces cycles of necrosis and promotes more effective muscle repair leading to decreased fibrosis. There is no data that demonstrates reduced necrosis or effective muscle repair in mdx-CCR2 mice.*

Response: To address this point, we have added new data in which IgG staining was used to quantify muscle necrosis (Weller et al, 1990) on diaphragm tissue sections. Using this approach, the mdx group showed a higher level of necrosis compared to mdx-CCR2-/- at 12 weeks of age, which is the same time point at which we demonstrate a reduction in fibrosis (no differences were found at 6 weeks, similar to the data on % central nucleation). These data are now shown in panel I of Fig. 7 in the revised manuscript.

9. *It would be helpful to provide information on the sex, whether animal were randomized before anti-CCR2 treatment. Was the data acquired in a blinded fashion?*

Response: All animals used for the experiments were males and assigned randomly to the treatment or control groups. In addition, data from the two groups were collected and analysed in a blinded fashion. These details are now provided in the revised methods section.

10. It would be helpful to know that pharmacological anti-CCR2 treatment actually blocked the CCR2. If so what is the effect of treatment on MO/MQ infiltrates in the skeletal muscle? What is the effect of treatment on fibrosis?

Response: The effects of anti-CCR2 fusokine treatment on macrophage infiltration in the muscle are shown in panel B of Fig. 9 in the revised manuscript: F4/80 immunostaining revealed a significantly lower macrophage content in fusokine-treated diaphragms versus controls ($p < 0.001$). The effects of anti-CCR2 fusokine treatment on muscle fibrosis are shown in panel F of Fig. 9: Gomori's modified Trichrome staining demonstrated that fusokine-treated diaphragms had significantly less fibrosis than controls ($p < 0.05$).

11. *Specific force (N/cm2) data (Figure 9F) indicates that controls generates <10N/cm2 at all frequencies. On the other hand mdx mice general show >10N/cm2 after 50Hz (Figure 8 A & B). Please clarify?*

Response: In Fig. 8, the mean maximal force generation values are about 12 N/cm2 at 6 weeks of age and 10 N/cm2 at 12 weeks of age; this is consistent with some degree of disease worsening between 6 and 12 weeks. In the original Fig. 9F (now Fig. 9G in the revised manuscript), the mean maximal force generation value is also approximately 10 N/cm2 at 9 weeks of age in control (non-fusokine treated) mdx diaphragms. As shown below, the force-frequency curves for these groups are essentially identical except for the youngest mdx age group which exhibits a slightly higher force generation during 100 Hz stimulation. Therefore, the specific force data are all internally consistent with one another.

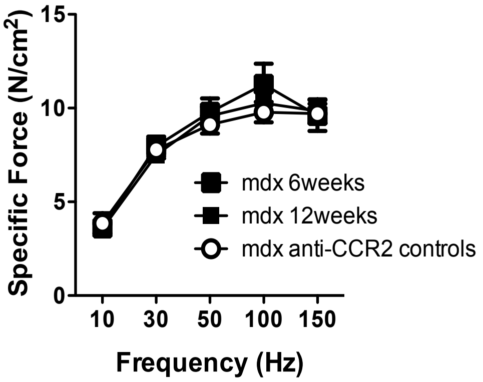

References Cited

Aragay AM, Mellado M, Frade JM, Martin AM, Jimenez-Sainz MC, Martinez-A C, Mayor F (1998) Monocyte chemoattractant protein-1-induced CCR2B receptor desensitization mediated by the G protein-coupled receptor kinase 2. *Proc Natl Acad Sci U S A* **95:** 2985-2990

Cabrera S, Gaxiola M, Arreola JL, Ramírez R, Jara P, D'Armiento J, Richards T, Selman M, Pardo A (2007) Overexpression of MMP9 in macrophages attenuates pulmonary fibrosis induced by bleomycin. *Int J Biochem Cell Biol* **39:** 2324-2338

Graham DJ (2009) D6 and the atypical chemokine receptor family: novel regulators of immune and inflammatory processes. *Eur J Immunol* **39**:342-51

Karpati G, Carpenter S, Prescott S (1988) Small-caliber skeletal muscle fibers do not suffer necrosis in mdx mouse dystrophy. *Muscle Nerve* **11:** 795-803

Onozuka I, Kakinuma S, Kamiya A, Miyoshi M, Sakamoto N, Kiyohashi K, Watanabe T, Funaoka Y, Ueyama M, Nakagawa M, Koshikawa N, Seiki M, Nakauchi H, Watanabe M (2011) Cholestatic liver fibrosis and toxin-induced fibrosis are exacerbated in matrix metalloproteinase-2 deficient mice. *Biochem Biophys Res Commun* **406:** 134-140

Radbill BD, Gupta R, Ramirez MC, DiFeo A, Martignetti JA, Alvarez CE, Friedman SL, Narla G, Vrabie R, Bowles R, Saiman Y, Bansal MB (2011) Loss of matrix metalloproteinase-2 amplifies murine toxin-induced liver fibrosis by upregulating collagen I expression. *Dig Dis Sci* **56:** 406-416

Vetrone SA, Montecino-Rodriguez E, Kudryashova E, Kramerova I, Hoffman EP, Liu SD, Miceli MC, Spencer MJ (2009) Osteopontin promotes fibrosis in dystrophic mouse muscle by modulating immune cell subsets and intramuscular TGF-beta. *J Clin Invest* **119:** 1583-1594

Weller B, Karpati G, Carpenter S (1990) Dystrophin-deficient mdx muscle fibers are preferentially vulnerable to necrosis induced by experimental lengthening contractions. *J Neurol Sci* **100:** 9-13

© EMBO

2nd Editorial Decision 13 August 2014

Thank you for the submission of your revised manuscript to EMBO Molecular Medicine. We are sorry that it has taken longer than usual to get back to you on your manuscript. We experienced delays in securing the re-evaluations and I also wished to discuss this case with an external expert, who was not immediately available, and the Chief Editor.

As you will see, fundamental concerns remain that preclude publication of the manuscript in EMBO Molecular Medicine.

While Reviewer 1 is now positive, Reviewer 2 remains quite critical and notes that the new information concerning the genetic makeup of the mice used in the study raises issues that are not addressable without substantial experimentation. We do not consider this concern to be further reaching with respect to his/her previous evaluation, because it is an opinion based on information that was not available in the first version of your manuscript. Reviewer 2 is also puzzled by the histology readouts.

As mentioned above, I further consulted with an external advisor with strong specific expertise. S/he agreed with Reviewer 2 and mentioned specifically that strain heterogeneity may not only result in different macrophage numbers, but also in force differences thus potentially compromising comparative analysis between wild-type, dystrophic and treated animals, especially considering the limited extent of the experimental outcomes. The Advisor was also not convinced of the quality and depth of analysis of the histological analysis.

I hope that you understand that, also considering our policy to allow a single round of revision only (except for minor amendments), we have no choice but to return the manuscript to you at this stage so that you may consider an alternative venue for your work. In our assessment it is not realistic to expect you to be able to address these issues experimentally in a reasonable time frame.

I am sorry to have to disappoint you at this stage. I hope that the Reviewer evaluations will be helpful in your continued work in this area.

***** Reviewer's comments *****

Referee #1 (Remarks):

The authors answered to all the concerns I raised and provided new experimental data that improve the manuscript.

Referee #2 (Remarks):

Authors addressed most of the comments. In response to the Question #1 in the previous review authors now indicate genetic backgrounds of mouse strains used in this study (CCR2-/- mice on C57BL6; mdx mice on C57BL10; mdx-CCR2-/- mice on mixed background and WT controls are on C57BL10 background).

It is important to rule out that the data presented in this paper is not due to strain differences but due to the absence of CCR-2. One way to rule out genetic background differences is to backcross CCR2-/- (BL6) mice onto mdx background (BL10). Obviously this takes time and effort but it gives reliable and reproducible phenotype. Next reasonable alternative is to use WT mixed (BL6 and BL10) background mice as controls. In my view proper controls are critical to interpret mdx-CCR-/- data presented in this paper.

I am also concerned with muscle fiber size data presented in figure 7F and 7G. Fiber size variation is one of the main features of mdx pathology. Published literature indicates that mdx mice generally have broader spread of fiber size distribution than WT mice. The data presented Figure 7 indicates that most of the mdx fibers are smaller than BL10 WT mice and there are no bigger muscle fibers.

Appeal 25 August 2014

Thank you for your letter. After carefully considering the points you raised in your decision, I am concerned that the editors as well as Reviewer 2 have lost sight of the main substance of our paper and have been unduly distracted by the mixed genetic background of the mdx-CCR2-/- mice and lack of abnormally large fibers observed in the mdx diaphragm. We strongly believe both of these points are easily addressed and certainly do not justify the rejection of this paper, which contains a tremendous amount of novel information with substantial translational potential for Duchenne muscular dystrophy patients.

The notion that our findings of dramatically altered macrophage recruitment in mdx-CCR2-/-mice can be explained by mouse background strain differences between BL6 and BL10, rather than the major evident fact that the mice are lacking CCR2 which is already well known to be a central regulator of macrophage recruitment, is an extremely unlikely hypothesis. In fact, a previous study by Beastrom N et al (Am J Pathol 179:2464-74, 2011) has directly compared mdx mice on the BL6 and BL10 backgrounds, and found no differences in the same parameters we measured in our study: infiltration by macrophages and lymphocytes, hydroxyproline content, myofiber damage, or limb muscle ex vivo contractile force at the same age (12 weeks). Interestingly, they did find lower ex vivo contractile force values in the diaphragms of mdx mice on the BL6 background, which makes it even more implausible to propose that the improvements we find in mdx-CCR2-/- diaphragms can be attributed to the minority (25%) BL6 component of their genetic background. Furthermore, in the other model system used in our study, which consisted of pharmacological inhibition of CCR2, the treated and untreated mdx mice were littermates and hence had exactly the same genetic background. Therefore, pharmacological experiments in mice with identical genetic background also revealed the same result: interference with CCR2 function improved dystrophic muscle histology and muscle strength.

The genetic background of the mdx-CCR2-/- mice of our study is predominately BL10 (75%), and for this reason we used BL10 mice as wild-type controls. To specifically respond to the issues raised by your external advisor, we have provided data below which show that there are no differences in either force generation or macrophage numbers between BL6 and BL10 mice. These data are consistent with the previously mentioned paper by Beastrom N et al., which also compared these parameters in the two wild-type mouse strains and reported the same findings. Investigators in the muscular dystrophy field routinely use both BL6 (examples: Vallese D et al. The Rag2- Meakins-Christie Laboratories IL2rb Dmd mouse: a novel dystrophic and immunodeficient model to assess innovating therapeutic strategies for muscular dystrophies. Molecular Therapy 21:1950-7, 2013; Long C et al. Prevention of muscular dystrophy in mice by CRISPR/Cas9-mediated editing of germline DNA. Science epub ahead of print 2014) and BL10 (eg., Henrique-Pons et al. Role of toll-like receptors in the pathogenesis of dystrophin-deficient skeletal and heart muscle. Human Molecular Genetics 23:2604-17, 2014; Hao S et al. Improved regenerative myogenesis and muscular dystrophy in mice lacking Mkp5. Genes and Development 22:1747-52, 2008) as wild-type controls for genetically modified mdx mice.

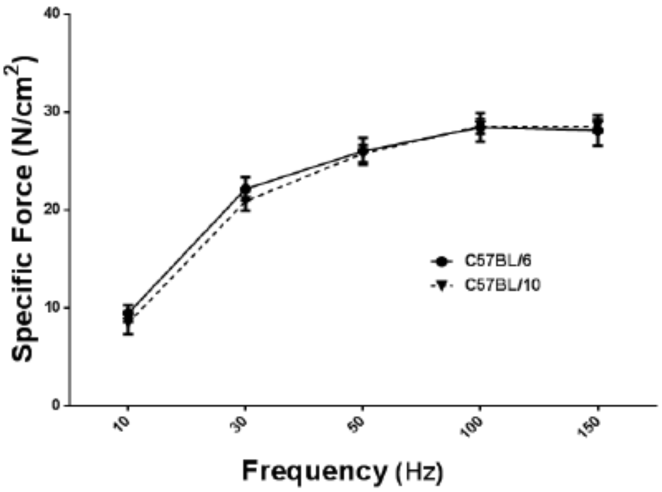
Specific Force (N/cm2)
40
30
20
10
0
10
30
50
100
150
Frequency (Hz)
C57BL/6
C57BL/10

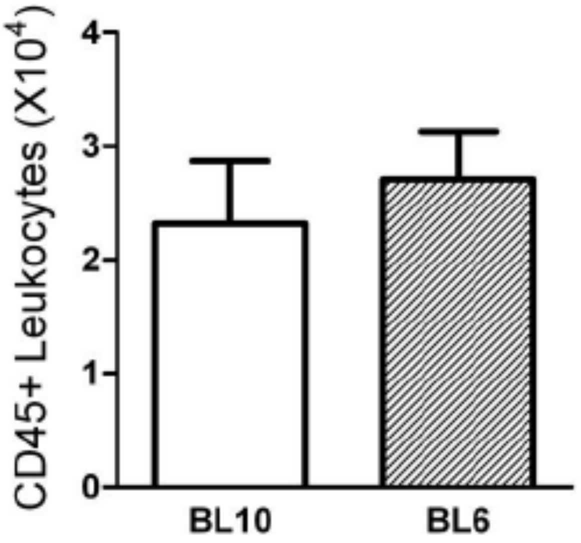
CD45+ Leukocytes (X10^4)
4
3
2
1
0
BL10
BL6

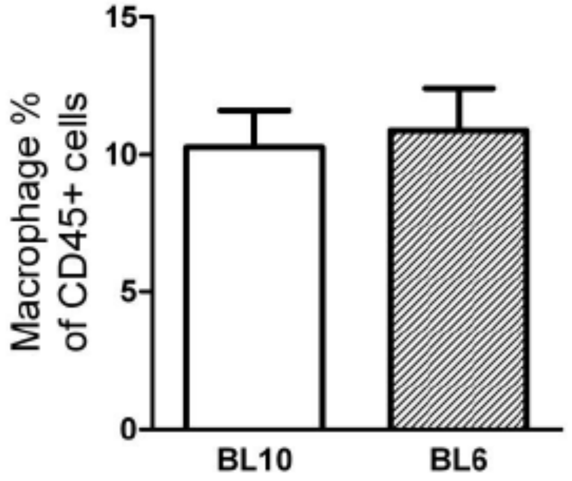
Macrophage %
of CD45+ cells
15
10
5
0
BL10
BL6

We are puzzled by the statement that your advisor is not satisfied with the depth of histological analysis. The histological outcomes which we have shown (central nucleation, fiber size, necrosis, inflammatory cells and fibrosis) are comprehensive and totally in line with the current histological outcomes recommended for pre-clinical studies in mdx mice (Grounds MD et al. Neurobiol Dis 31:1-19, 2008). Furthermore, from a clinical translation point of view, the most important observation of our study is that CCR2 deficiency improves diaphragm force generation and functional resistance to contraction-induced injury, which many studies in the field have failed to assess. In this sense our study goes beyond routine histological outcomes to determine what actually matters to the muscular dystrophy patient, which is contractile function of the essential skeletal muscle responsible for survival, the diaphragm.

Finally, Reviewer 2 raised a concern about our muscle fiber size distribution data in the diaphragm, stating that "published literature indicates that mdx mice generally have a broader spread of fiber size distribution than wild-type mice" and wondering why we observe mdx fibers which are smaller than wild-type but "no bigger muscle fibers". The reviewer is apparently not aware of the fact that the mdx diaphragm differs from mdx limb muscles in this respect. In the mdx diaphragm, fibers are smaller than wild-type and there is no increase in the percentage of larger fibers - this observation is not new and was published many years ago by Louboutin JP et al. (Neuromusc Disord. 3:463-69, 1993). Therefore, this point is easily addressed and there is no reason for concern as our data are entirely in line with the published literature.

In summary, we feel that the concerns raised in your letter can be addressed by further explanation or clarification and are certainly not of sufficient importance to warrant rejection of this paper. There are several novel and significant findings in our paper, which together represent a timely and important conceptual scientific breakthrough with significant translational potential. We believe that a careful reading of our manuscriptwill demonstrate that it is fundamentally contributing to our understanding of macrophages and how their differential functions affect Duchenne muscular dystrophy.

We strongly believe that this manuscript merits reconsideration by the editors.
Thank you in advance for your consideration,

3rd Editorial Decision 03 September 2014

Thank you for your letter asking us to re-consider our previous decision on your manuscript. We have now carefully evaluated your arguments and sought additional advice on how to proceed. In light of your arguments and provision of the added experiments in BL6 and BL10 mice, and together with our additional advice, we are happy to let you know that we will be able to accept your manuscript pending the following final amendments:

We would like to encourage you to add as supplementary information the data provided on force and immune cell numbers in BL6 and BL10 while nevertheless mentioning the current limitations of your mouse model according to the different background used in control mice. Please make sure to amend the methods section by indicating the number of times CCR2-/- mice were back-crossed with mdx mice and the resulting genetic background

Please submit your revised manuscript within two weeks. I look forward to seeing a revised form of your manuscript as soon as possible.

Additional Author Correspondence 04 September 2014

Thank you for this very good news. We greatly appreciate the care and diligence of the editors. We certainly will make all of the changes you have suggested and return the manuscript to you within the next 2 weeks.

2nd Revision - authors' response 16 September 2014

We have completed the amendments you have requested and uploaded the revised manuscript and figures. Specifically we have included the data on force and immune cell numbers of BL10 and BL6 control mice in the supplementary data, and indicated in the methods section the breeding strategy leading to different genetic backgrounds of the mice.

We appreciate the attention you and your editorial staff have shown to this manuscript and we hope the paper is now acceptable for publication.
