# Supplementary figures and images for: Inflammatory monocytes promote progression of Duchenne muscular dystrophy and can be therapeutically targeted via CCR2
Source: EMBO Mol Med. 2014 Oct 13;6(11):1476–92. doi: 10.15252/emmm.201403967 (PMC4237472; doi:10.15252/emmm.201403967)

Figure S1

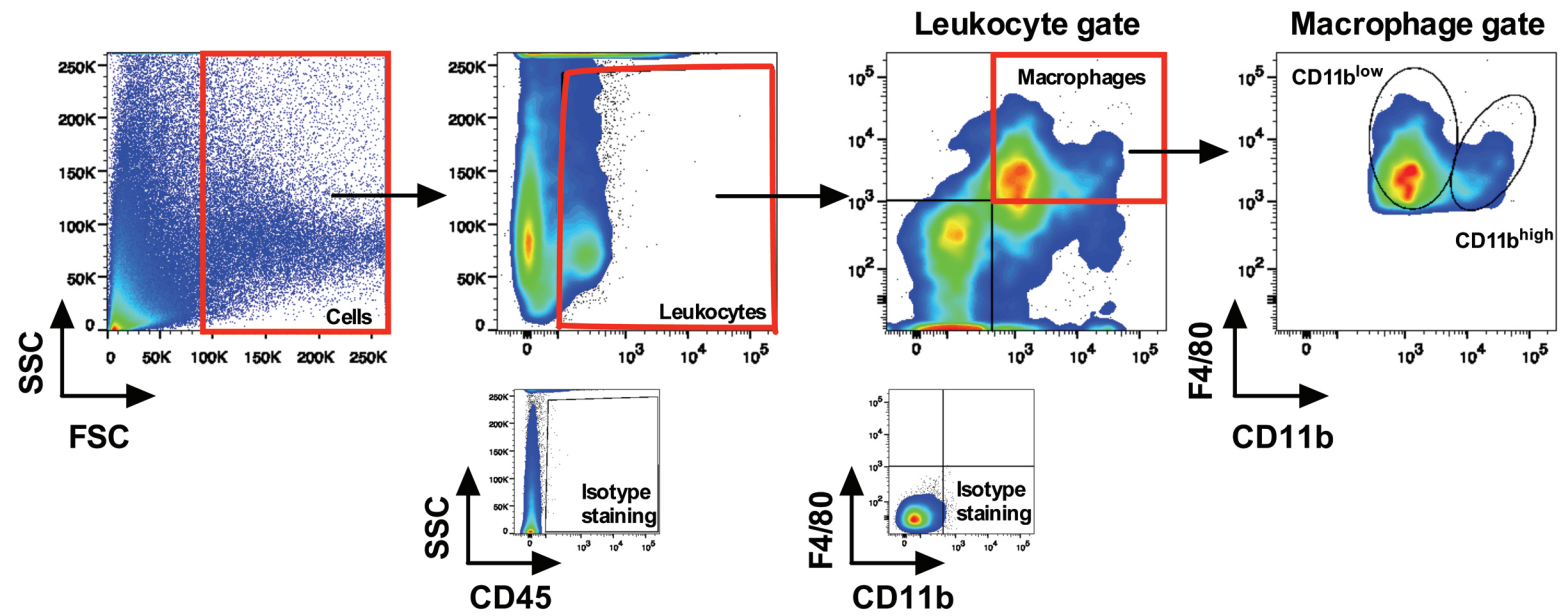

Supplement: Supplementary file 2 [file emmm0006-1476-sd2.pdf]

Figure S2

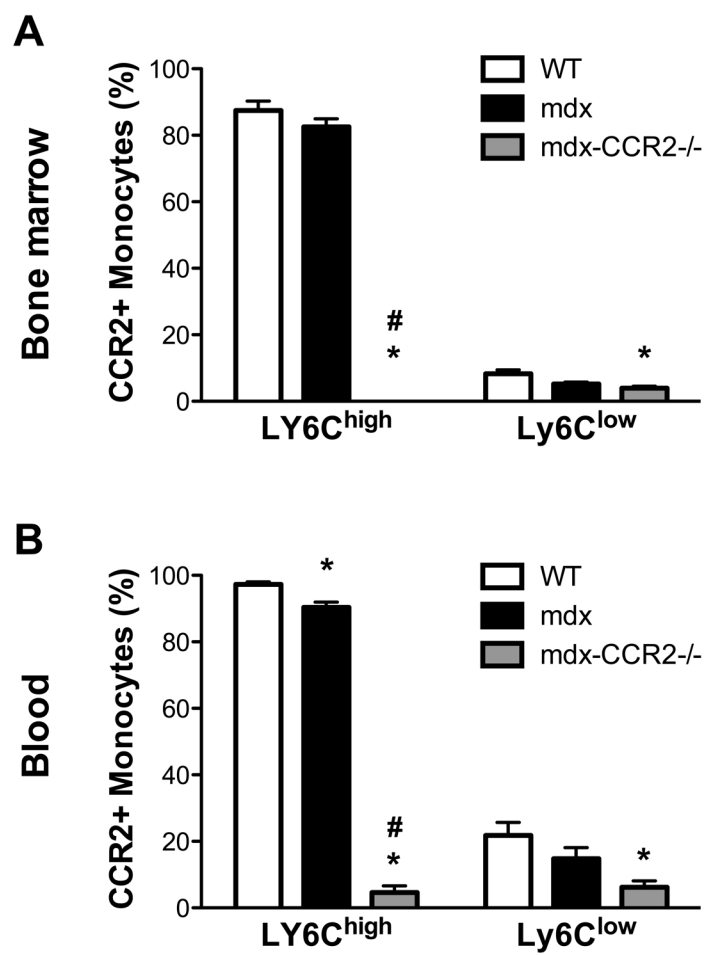

Supplement: Supplementary file 3 [file emmm0006-1476-sd3.pdf]

Figure S3

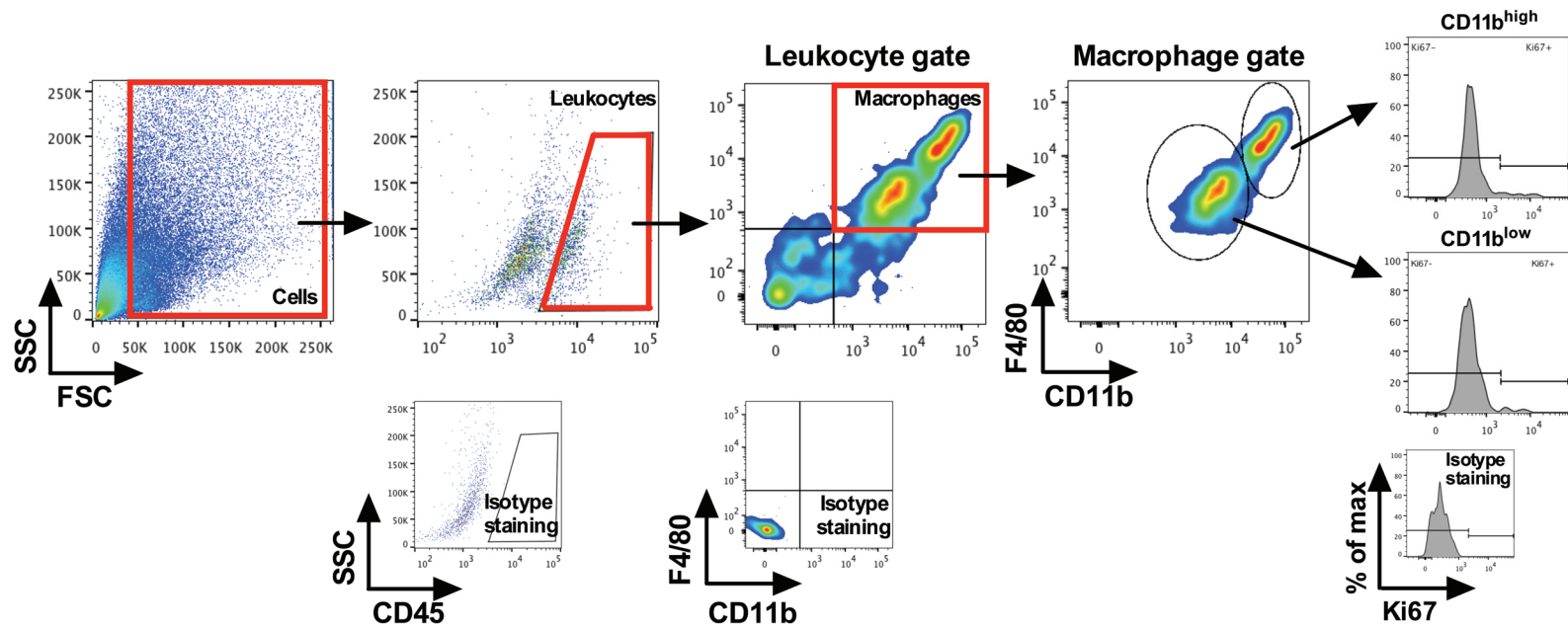

Supplement: Supplementary file 4 [file emmm0006-1476-sd4.pdf]

Figure S4

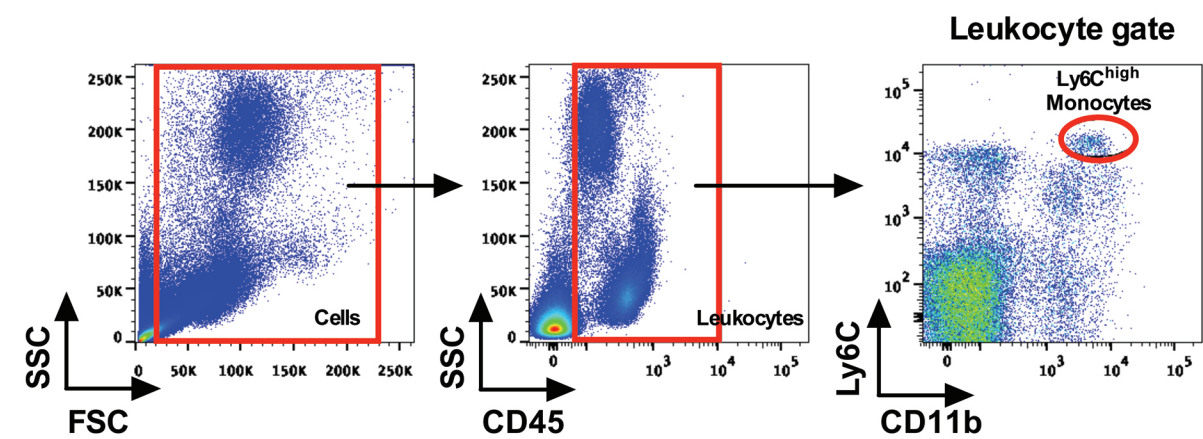

Supplement: Supplementary file 5 [file emmm0006-1476-sd5.pdf]

Figure S5

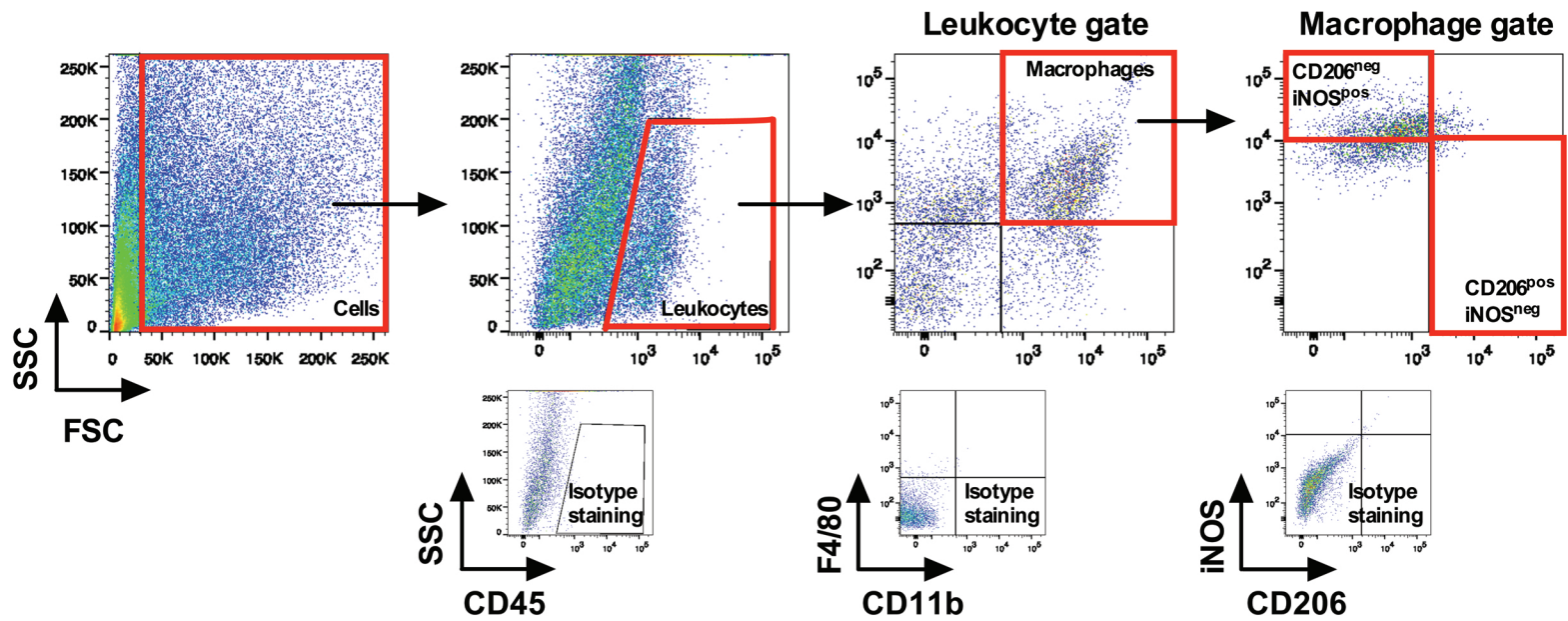

Supplement: Supplementary file 6 [file emmm0006-1476-sd6.pdf]

Figure S6

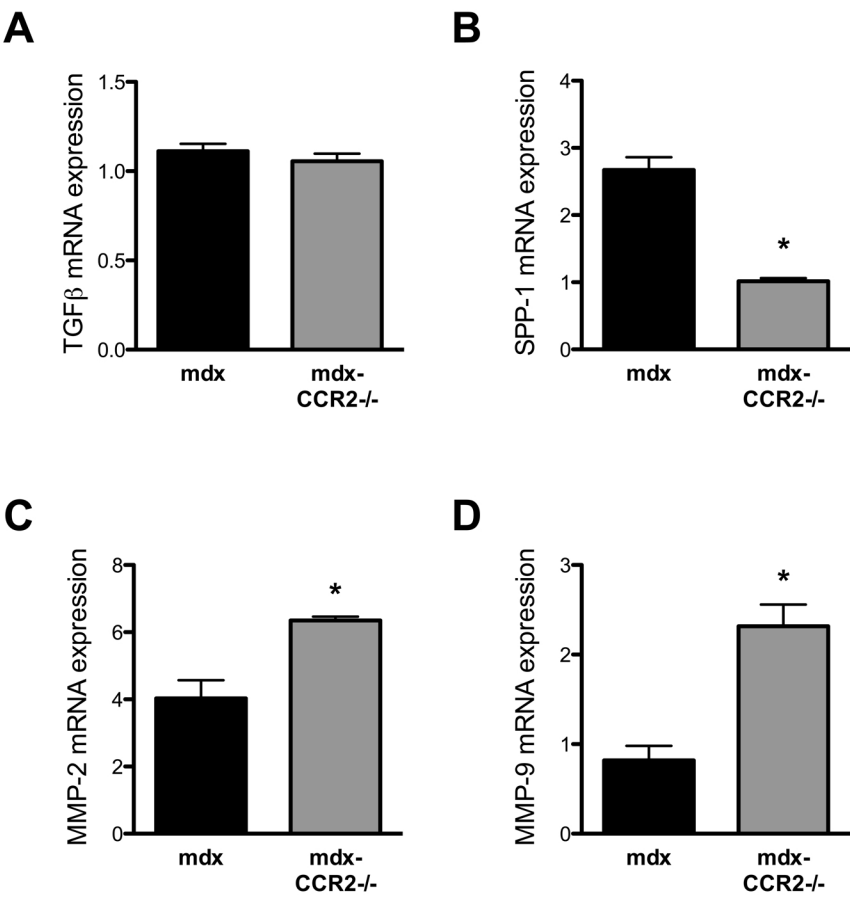

Supplement: Supplementary file 7 [file emmm0006-1476-sd7.pdf]

**Figure S7**

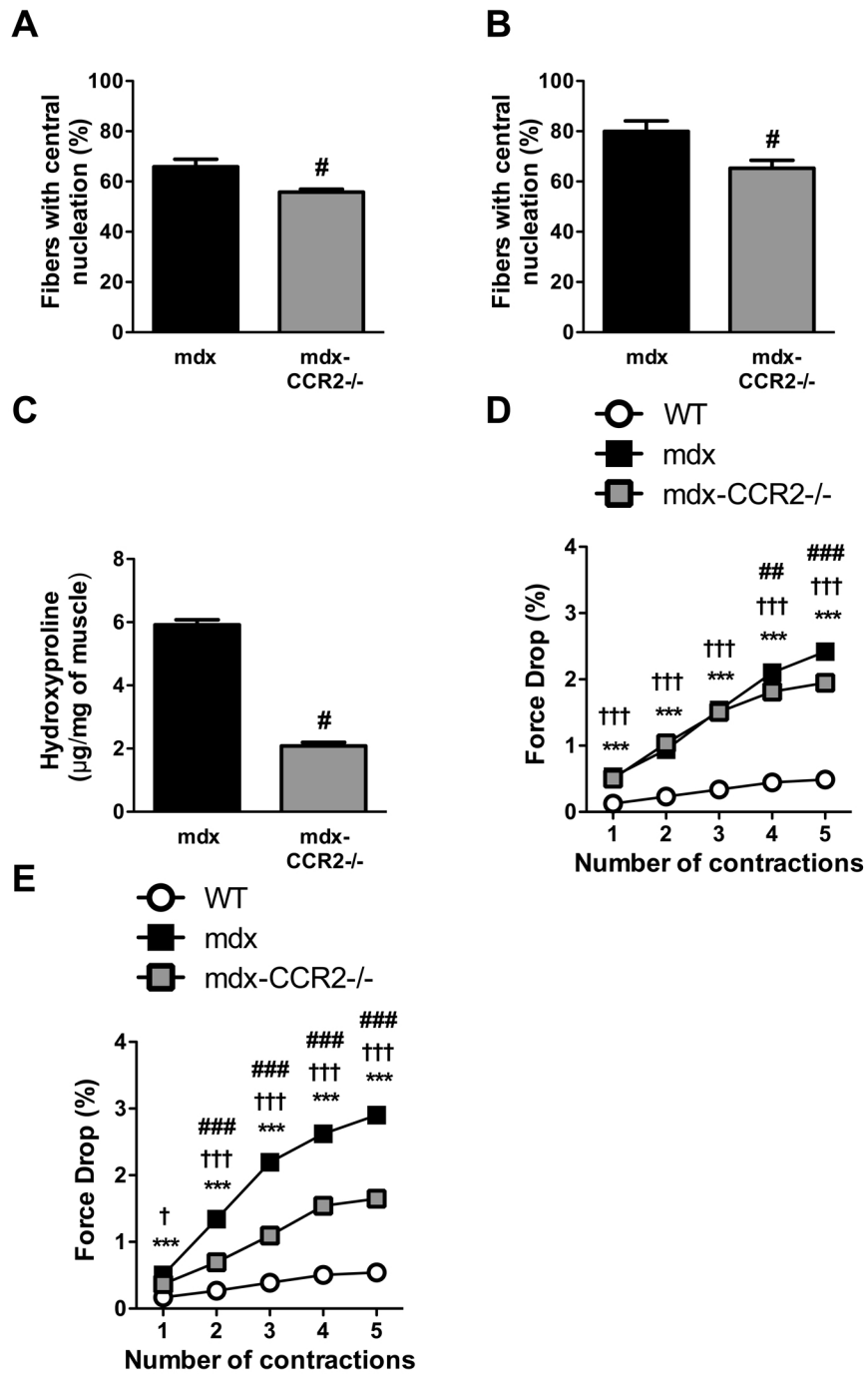

Supplement: Supplementary file 8 [file emmm0006-1476-sd8.pdf]

Figure S8

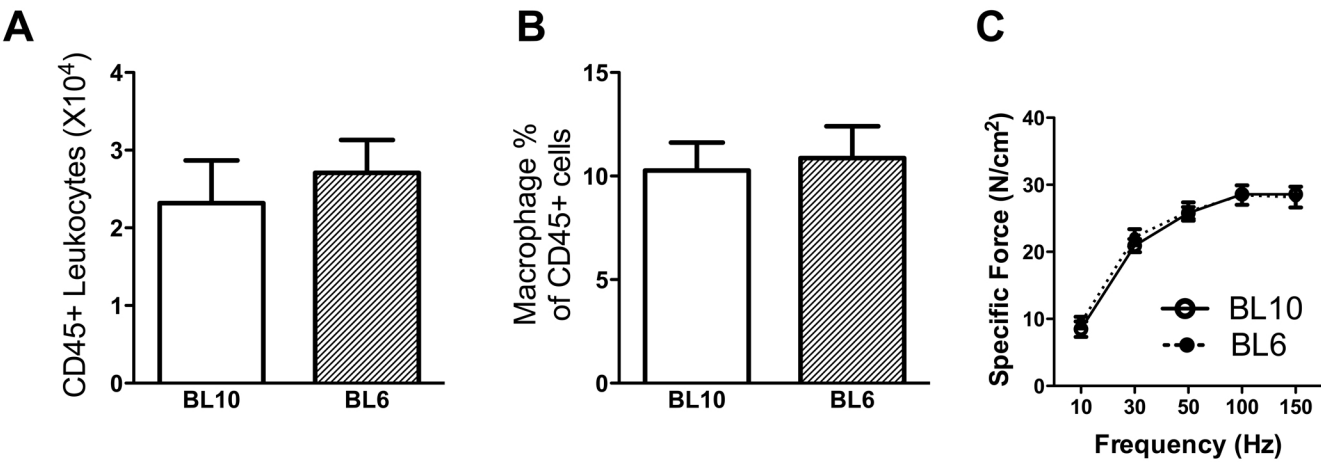

Supplement: Supplementary file 9 [file emmm0006-1476-sd9.pdf]
