# Supplementary material for: Inflammatory monocytes promote progression of Duchenne muscular dystrophy and can be therapeutically targeted via CCR2
Source: EMBO Mol Med. 2014 Oct 13;6(11):1476–92. doi: 10.15252/emmm.201403967 (PMC4237472; doi:10.15252/emmm.201403967)
Supplement: Supplementary file 10 [file emmm0006-1476-sd10.pdf]

Supplementary Table S1. Primer Sequences

Gene	Sequence (5' to 3')
Dystrophin	F AACTCATCAAATATGCGTGTTAGTG<br>R GTCACTCAGATAGTTGAAGCCATTTAA (mutated)<br>R GTCACTCAGATAGTTGAAGCCATTTAG (wild type)
CCR2	F CTTGGGTGGAGAGGCTAT TC (wild type)<br>R AGGTGAGATGACAGGAGATC (wild type)
	F CCACAGAATCAAAGGAAATGG (mutated)<br>R CCAATGTGATAGAGCCCT GTG (mutated)
TGFβ	F TGCGCTTGCAGAGATTAA<br>R CGTCAAAAGACAGCCACT
SPP-1	F GATGATGATGACGATGGAGACC<br>R CGACTGTAGGGACGATTGGAG
MMP-2	F CCCGCAAGCCCAAGTGGGAC<br>R AAGCGCAGCGGAGTGACGTC
MMP-9	F GTTCGGCCATGCACTGGGCTT<br>RGGGTCAGGCTTAGAGCCACGAC
