# Supplementary material for: Inflammatory monocytes promote progression of Duchenne muscular dystrophy and can be therapeutically targeted via CCR2
Source: EMBO Mol Med. 2014 Oct 13;6(11):1476–92. doi: 10.15252/emmm.201403967 (PMC4237472; doi:10.15252/emmm.201403967)
Supplement: Supplementary file 11 [file emmm0006-1476-sd11.pdf]

SUPPLEMENTARY FIGURE LEGENDS

Fig. S1. ***Gating strategy to identify intramuscular macrophages.*** Forward versus side scatter plots were used to exclude cellular debris. Non-permeabilized leukocytes were selected based on their expression of CD45, and were plotted for CD11b and F4/80 expression. Within the CD11b+F4/80+ macrophage (MP) gate, cells were further classified as $CD11b^{high}$ and $CD11b^{low}$. Representative isotype staining is also shown.

Fig. S2. ***CCR2 expression on*** $Ly6C^{high}$ ***and*** $Ly6C^{low}$ ***monocytes.*** Monocytes were identified as $CD11b+Ly6C^{high}$ or $CD11b+Ly6C^{low}$ and CCR2 expression on both populations was assessed. Percentage of $Ly6C^{high}$ and $Ly6C^{low}$ monocytes expressing CCR2 in **(A)** Bone marrow, * comparison to WT: P=5.4e-9 (for $Ly6C^{high}$) and P=0.008 (for $Ly6C^{low}$); # comparison between mdx and mdx-CCR2-/-: P=5.4e-9; and **(B)** Blood, * comparison to WT: P=0.003 (for mdx $Ly6C^{high}$), P=5.8e-9 and P=0.03 (for mdx-CCR2-/- $Ly6C^{high}$ and $Ly6C^{low}$ respectively); # comparison between mdx and mdx-CCR2-/-: P=5.8e-9. Data are group means ± SE (WT n=8; mdx n=6; mdx-CCR2-/- n=6).

Fig. S3. ***Gating strategy to identify proliferating intramuscular macrophages.*** Following exclusion of cellular debris based on forward and side scatter plots, CD45 expression was used to gate permeabilized leukocytes. Infiltrating MPs were identified by their expression of CD11b and F4/80 and characterized as $CD11b^{high}$ or $CD11b^{low}$ based on their CD11b expression level. Ki67 expression was then assessed on the two MP populations.

Fig. S4. ***Gating strategy to identify inflammatory monocytes.*** Cells gated based on their forward and side scatter properties were plotted for CD45 expression to identify leukocytes. Inflammatory monocytes were identified as CD11b+ with high Ly6C expression. Representative plots from blood staining are shown, and a similar gating approach was used in bone marrow and spleen.

Fig. S5. ***Gating strategy to characterize polarization phenotype of diaphragm-infiltrating macrophages.*** Cellular debris was excluded based on forward and side scatter properties, followed by plotting cells for CD45 expression to gate leukocytes. MPs were identified based on dual expression of CD11b and F4/80. Cells from the MP gate were then plotted for iNOS and CD206, where MPs were characterized as $CD206^{neg}$ $iNOS^{pos}$ and $CD206^{pos}$ $iNOS^{neg}$.

Fig. S6. ***Quantification of pro- and anti-fibrotic factors in bone marrow derived MPs.*** Transcript levels of pro-fibrotic **(A)** TGF-beta and **(B)** Osteopontin/SPP-1 (* P=0.0001), in bone marrow derived MPs from mdx and mdx-CCR2-/- mice. Transcript levels of anti-fibrotic **(C)** MMP (matrix metalloproteinase)-2 (* P=0.006) and **(D)** MMP-9 (* P=0.002) in the same groups are also shown. Data are group means ± SE (n=4 mice per group, 2 independent experiments).

Fig. S7. ***CCR2 ablation decreases dystrophic pathology in tibialis anterior (TA) muscle.*** The percentage of centrally nucleated fibers in the TA limb muscle was reduced in mdx-CCR2-/- mice at **(A)** 6 weeks (# P=0.003) and **(B)** 12 weeks (# P=0.02) compared to age-matched mdx mice. **(C)** Hydroxyproline content of TA muscles was also reduced in mdx-CCR2-/- mice at 12 weeks (# P=1.3e-8). Eccentric contraction-

induced force drop in the mdx-CCR2-/- TA was less pronounced than in mdx mice at **(D)** 6 weeks and particularly at **(E)** 12 weeks of age (6 weeks: n=8 in each group; 12 weeks: WT n=8, mdx n=7, mdx-CCR2-/- n=7; three independent experiments). In panels D & E, *** P<0.001 for WT compared to mdx; † P<0.05, ††† P<0.001 for WT compared to mdx-CCR2-/-; # P<0.05, ## P<0.01, ### P<0.001 for mdx compared to mdx-CCR2-/-.

Fig. S8. ***Macrophage content and force-generating capacity in BL10 and BL6 mice.*** Total muscle CD45+ leukocyte counts **(A)** and muscle MPs as a percentage of the leukocytes **(B)** in age-matched BL10 (n=3) and BL6 mice (n=5). **(C)** Force-frequency curves during electrical stimulation of diaphragm muscle from age-matched BL10 (n=8) and BL6 (n=10) mice**.** Data are group means ± SE, no significant differences in the above parameters between groups.
